# Cellular senescence defining the disease characteristics of Crohn’s disease

**DOI:** 10.3389/fimmu.2025.1616531

**Published:** 2025-06-30

**Authors:** Wenyu Zhang, Xianzong Ma, Wenqing Tian, Yongsheng Teng, Meihua Ji

**Affiliations:** ^1^ School of Nursing, Capital Medical University, Beijing, China; ^2^ Senior Department of Gastroenterology, The First Medical Center of Chinese PLA General Hospital, Beijing, China; ^3^ Department of Gastroenterology, The Seventh Medical Center of Chinese PLA General Hospital, Beijing, China; ^4^ Department of Gastroenterology, Chongqing University Cancer Hospital, Chongqing, China; ^5^ Department of Gastroenterology, Chongqing General Hospital, Chongqing University, Chongqing, China

**Keywords:** Crohn’s disease, cellular senescence, machine learning, immune infiltration heterogeneity, biomarker

## Abstract

**Background:**

Crohn’s disease (CD) is a complex and heterogeneous inflammatory disease whose most important feature is immune dysregulation. As a basic cell response, cellular senescence (CS) can regulate the immune response involved in a variety of inflammatory diseases. However, the role of CS in the pathogenesis and diagnosis prediction of CD are still unknown.

**Methods:**

We utilized CD-related datasets from the GEO database for differential gene expression analysis, and CS related differentially expressed genes (CSRDEGs) in CD by a comprehensive bioinformatics analysis encompassing GSEA, WGCNA, and various interaction networks. The support vector machine (SVM) algorithm, random forest algorithm and LASSO regression analysis was used to construct a diagnostic model. And based on CSRDEGs, we further constructed a Cellular senescence score (CSscore) model. Different disease subtypes (cluster1/cluster2) were identified by the consensus clustering method. The assessment of immune cell infiltration and its correlation with CSRDEGs was analyzed by ssGAEA and CIBERSORT.

**Results:**

We identified 10 hub CS related differentially expressed genes (CSRDEGs) in CD. Based on CSRDEGs, we further constructed a diagnostic model (AUC = 0.880) containing 5 CSRDEGs (*CDKN1A*, *IL1A*, *PML*, *SIRT1*, and *STAT3*) through machine learning algorithm and other methods and analyzed the correlation with immune cell infiltration. In addition, a CS Scores model (Low or High) based on the 7 CSRDEGs (*CDKN2B*, *IGFBP7*, *IL1A*, *IL6*, *PML*, *SIRT1*, and *STAT3*) shows different characteristics, reaffirming the inflammatory regulatory role of CS in CD. Finally, the subtype construction (cluster1 and cluster2) based on 10 CSRDEGs shows the heterogeneity of the disease and affirms that CS is a prominent feature of CD.

**Conclusions:**

These results suggest that CS is an important feature of CD, and CSRDEGs can be used to construct disease diagnostic models and distinguish disease subtypes. Further investigation of the mechanism of immune dysregulation caused by CS can deepen our understanding of the pathogenesis of CD.

## Introduction

Crohn’s disease (CD), a chronic and debilitating form of inflammatory bowel disease (IBD), can affect any part of the gastrointestinal tract. Its clinical course is progressive and destructive (marked by alternating phases of inflammatory flares and remission), however, the exact pathogenic mechanism is still unclear (1, 2). Recognized by the World Health Organization (WHO) as a “modern refractory disease” and colloquially termed “green cancer” (3), CD has witnessed escalating incidence rates in industrialized regions, imposing substantial socioeconomic burdens (4, 5). While current treatments, including immunosuppressants, biologics, and surgical interventions, provide symptomatic relief, their efficacy is constrained by high relapse rates (6), significant adverse effects, and particularly poor responsiveness in structuring (B2) and penetrating (B3) phenotypes (7, 8). This underscores the urgent need to explore disease heterogeneity at the molecular level to identify novel therapeutic targets.

Previous studies have indicated that cellular senescence plays a critical role in various chronic inflammatory diseases (9, 10). Characterized by irreversible cell cycle arrest triggered by DNA damage or oxidative stress (10), senescent cells perpetuate tissue dysfunction through the senescence-associated secretory phenotype (SASP), releasing pro-inflammatory cytokines (e.g., IL-6, CXCL8), matrix metalloproteinases, and growth factors that exacerbate tissue damage and impair regeneration (11). Groundbreaking work by Tindle et al. (12) using patient-derived organoids (PDOs) and multi-omics profiling has redefined CD heterogeneity, identifying two molecular subtypes: an immune-deficient infectious subtype (IDICD) and a senescence-driven fibrostenotic subtype (S2FCD). The S2FCD subtype (prevalent in 39% of CD cases) exhibits hallmark senescence features, including γ-H2AX foci accumulation and elevated SA-β-gal activity, coupled with fibrogenic gene signatures (e.g., BAMBI downregulation). Notably, whole-exome sequencing revealed enrichment of mutations in the DNA damage-YAP-IL18 pathway, mechanistically linking replicative senescence to stricturing complications (13). Functional assays demonstrated impaired barrier integrity in S2FCD PDOs, evidenced by reduced transepithelial electrical resistance (TEER) and increased FITC-dextran permeability in stricturing (B2) subtypes, suggesting senescence-mediated epithelial dysfunction as a critical driver of fibrotic progression. Recent reports have posited that chronic inflammation is an endogenous factor driving cellular senescence, with inflammation-related molecular patterns promoting senescence, which in turn propagates further inflammation via SASP, thus establishing a vicious cycle (9). Immune cells play a pivotal role in recognizing and eliminating senescent cells; however, the effects of inflammation and SASP can lead to T cell pool dysfunction and chronic antigenic stimulation, precipitating premature senescence in immune cells. This results in immunosenescence, which consequently disrupts the structural integrity of immune organs and impairs immune response functions, diminishing the capacity to respond to infections and diseases, thereby increasing susceptibility to disease, enhancing inflammation, and elevating the risk for senescence-associated diseases (9, 14–16). Clinical data reveal a steady increase in the incidence of CD among elderly patients, coupled with poorer prognosis (17), further underscoring the pivotal role of senescence mechanisms in CD pathogenesis.

The pathogenesis of CD is extraordinarily complex, implicating intricate interactions among genetic, environmental, immunological, and gut microbiota factors (18). The intestinal immune system plays a vital role in defending against pathogenic invasions while maintaining immunological tolerance to gut microbiota; however, dysregulation of the intestinal immune system may precipitate the onset of CD (19). Research has demonstrated that regulatory T (Treg) cells in aged mice exhibit significantly diminished functionality in suppressing T conventional (Tconv) cell activity in IBD models, as well as reduced capacity to prevent Tconv cell senescence in radiation-induced senescent mouse models when compared to their younger counterparts (20). Recent investigations have shown a marked increase in the incidence of CD among the elderly with progressively severe implications for older patients (21). Additionally, studies suggest that cellular and immune-level senescence may correlate with CD pathology in aging populations (22). These findings suggest that senescence may play critical roles in modulating the progression of CD, and targeting senescence could open new avenues for the prevention and treatment of this disease in the future.

This study aims to identify hub biomarkers of cellular senescence related differentially expressed genes (CSRDEGs) and immune-related pathways associated with cellular senescence in CD through a comprehensive bioinformatics analysis encompassing differential expression analysis, Gene Set Enrichment Analysis (GSEA), Weighted Gene Co-expression Network Analysis (WGCNA), and the construction of various interaction networks. This multifaceted approach seeks to elucidate gene expression alterations and their underlying mechanisms in detail while identifying key regulatory factors, thereby providing a theoretical foundation for subsequent clinical applications.

## Methods

### Data download

**Figure 1:**
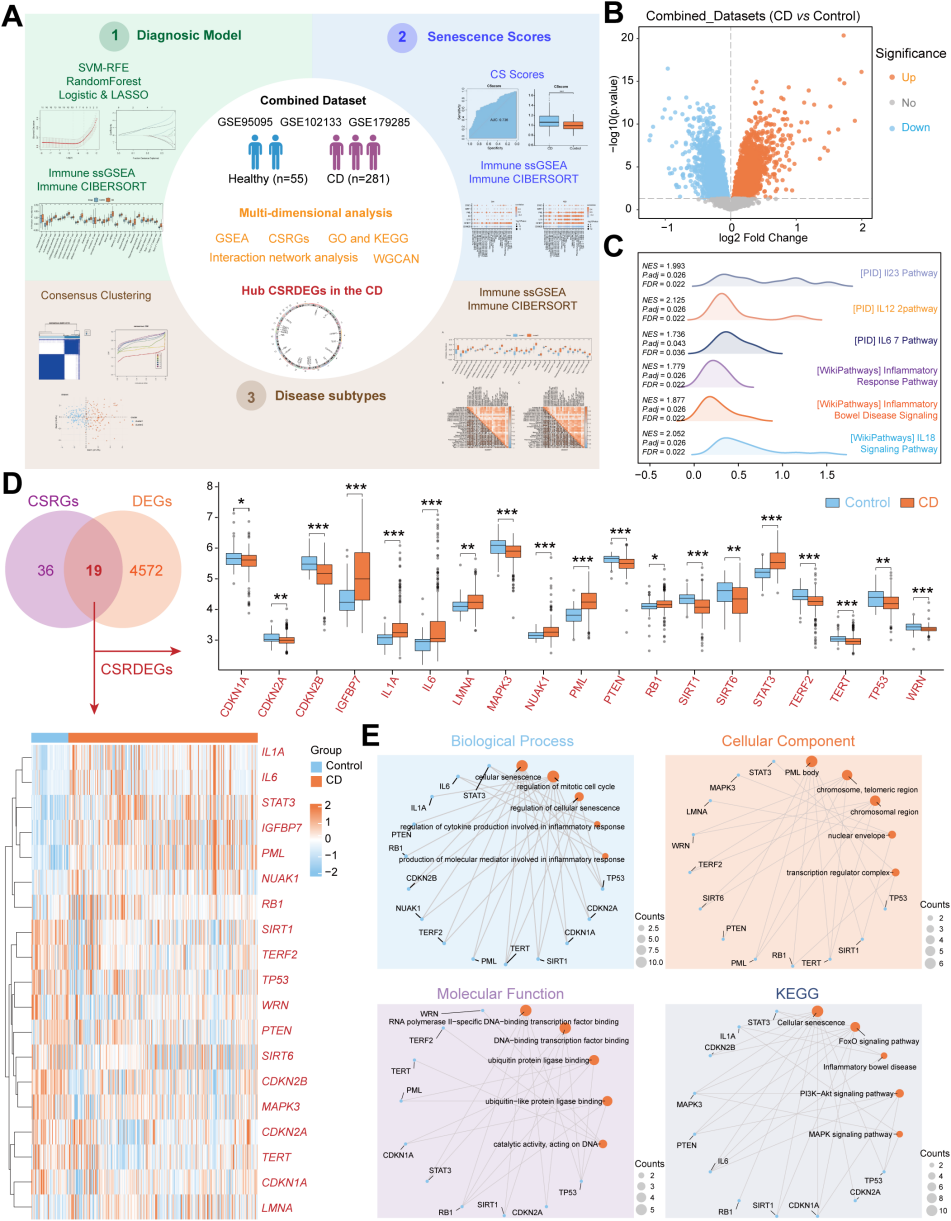
Identification and functional enrichment of CSRDEGs in CD. **(A)** Workflow of the study design. **(B)** The volcano plot of all DEGs (CD vs Control) in Combined dataset. **(C)** The ridge plot of GSEA enrichment analysis of all DEGs (CD vs Control). **(D)** Venn diagram of the intersection of the CSRGs and DEGs (CD vs Control). 19 CSRDEGs were found, and heatmap and box plot showed the expression of all the genes. **(E)** GO and KEGG network diagram analysis of the CSRDEGs. The blue dots represent CSRDEGs, and the orange dots represent specific pathways. CD, Crohn’s disease; GSEA, Gene Set Enrichment Analysis; CSRGs, Cellular senescence related genes; CSRDEGs, Cellular senescence related differentially expressed genes; GO, Gene Ontology; KEGG, Kyoto Encyclopedia of Genes and Genomes; WGCNA, weighted correlation network analysis; CSscore, Cellular senescence score; LASSO, least absolute shrinkage and selection operator; ssGSEA, single-sample gene-set enrichment analysis. *P < 0.05, **P < 0.01 and ***P < 0.001.

Gene expression profiles for CD were downloaded from the GEO database (23) (GSE95095, GSE102133 (24) and GSE179285 (25)). The sequencing platforms were was GPL14951 (GSE95095), GPL6244 (GSE102133) and GPL6480 (GSE179285). Sample composition was as follows: (1) GSE95095: 60 intestinal tissues (48 CD, 12 healthy controls); (2) GSE102133: 77 intestinal tissues (65 CD, 12 healthy controls); (3) GSE179285: 199 intestinal tissues (168 CD, 31 lesion-adjacent normal tissues) (see **Supplementary Table S1** for full details). All tissue samples were obtained from publicly available multicenter cohorts. We acknowledge that due to limitations in original metadata documentation: (1) specific biopsy locations (ileum vs. colon; inflammatory vs. non-inflammatory zones) were not uniformly available across samples, and (2) disease activity status (active vs. remission) could not be ascertained for all CD patients. Control samples primarily consisted of healthy intestinal tissues or macroscopically non-inflamed areas adjacent to lesions. Cellular senescence related genes (CSRGs) were retrieved from GeneCards database (https://www.genecards.org/) (26). yielding 55 protein-coding CSRGs (**Supplementary Table S2**, relevance score > 5). The study workflow is illustrated in **Figure 1A**.

### Identification of DEGs and CSRDEGs

Datasets (GSE95095, GSE102133, and GSE179285) were integrated to form a combined cohort comprising 281 Crohn’s disease (CD) samples and 55 normal control samples. To ensure data comparability and minimize technical artifacts arising from different platforms or batches, rigorous preprocessing was applied: (1) Probe Annotation: Probes were mapped to gene symbols using the annotation provided by the respective microarray platforms. (2) Batch Effect Correction: The removeBatchEffect function from the limma R package (version 4.1.2) (27) was utilized to explicitly model and remove potential batch effects associated with the different source datasets (GSE IDs). (3) Normalization: Between-array normalization was performed on the batch-corrected data using the normalizeBetweenArrays function (limma package), employing the default ‘quantile’ method to make expression distributions comparable across all samples. All subsequent analyses, including differential expression, visualization (e.g., heatmaps, box plots), and network construction, were performed exclusively on this preprocessed, batch-corrected, and normalized combined dataset.

Differential gene expression analysis between the CD group (n=281) and the control group (n=55) was conducted on the preprocessed combined dataset using the limma package. Genes exhibiting an absolute log_2_ fold-change (|logFC|) > 0 and an adjusted p-value (Benjamini-Hochberg (BH) method) < 0.05 were defined as Differentially Expressed Genes (DEGs). CSRDEGs were subsequently identified as the intersection between the DEG list and the CSRG list.

### GSEA, GO and KEGG enrichment analysis

The clusterProfiler package of R software was used to conduct GSEA (28) for all genes in the combined dataset, and perform Gene Ontology (GO) function enrichment analysis (29) and Kyoto Encyclopedia of Genes and Genomes (KEGG) pathway enrichment analysis (30) for CSRDEGs with screening criteria of adjusted P-value < 0.05 and value of false discover rate (FDR) (q.value) < 0.05.

### Construction of gene co-expression network

A co-expression network was constructed using the WGCNA R package (31) on the preprocessed combined dataset expression data. To focus the analysis on highly variable genes, genes ranked within the top 40% by variance across all samples were selected as input. An unsigned adjacency matrix was built using a soft-thresholding power (β) of 5, chosen based on the scale-free topology criterion to approximate a scale-free network (R² > 0.85). The adjacency matrix was transformed into a Topological Overlap Matrix (TOM), and hierarchical clustering with dynamic tree cutting was performed on the TOM-based dissimilarity to identify gene modules. The minimum module size was set to 60 genes. Modules exhibiting high similarity (dissimilarity < 0.20) were merged. Module eigengenes (MEs), representing the first principal component of each module, were calculated. The association between each module (represented by its ME) and the CD phenotype (coded as a binary trait: CD vs. Control) was quantified using Pearson correlation. Modules showing the strongest significant correlations (positive or negative) with CD status were selected as modules of interest. All genes within these significant modules were considered highly correlated with CD and designated as module feature genes.

### Construction of mRNA-drug, mRNA-TF, mRNA-miRNA and mRNA-RBP interaction networks

Comparative Toxicogenomics Database (CTD) (http://ctdbase.org/) (32) was used to predict potential drugs or small molecule compounds that interact with CSRDEGs; CHIPBase database (version 3.0) (https://rna.sysu.edu.cn/chipbase/) (33) and hTFtarget database (34) were used to find transcription factors (TF) that bind to CSRDEGs. ENCORI database (https://starbase.sysu.edu.cn/) (35) was used to predict miRNAs and RNA-binding proteins (RBP) that interact with CSRDEGs. Data visualization using Cytoscape software.

### Construction of CSRDEGs diagnostic model

In order to obtain the CSRDEGs diagnostic model of the Combined dataset, we employed the following workflow. First, support vector machine (SVM) model (36) was constructed using the expression matrix and grouping information (CD/Control). CSRDEGs were screened based on the number of genes yielding the highest accuracy and lowest error rate. Second, a random forest (RF) model (37) was built using the randomForest package based on the expression of these CSRDEGs. Third, a logistic diagnostic model was constructed using CSRDEGs meeting screening criteria (P-value < 0.05), with results visualized via Forest Plot. Fourth, Least Absolute Shrinkage and Selection Operator (LASSO) logistic analysis (38) was applied to these CSRDEGs using the glmnet package in R (parameters: set.seed (500), family = “binomial”) to construct Logistic-LASSO model.

$\text{risk}Score\;=\;\sum_{i}Coefficient\;\left(ERSRDEGs_{i}\right)*mRNA\;Expression\;\left(ERSRDEGs_{i}\right)$ (Coefficient__*i*_ denotes the regression weight for the *i*-th gene derived from LASSO analysis on the training set. Expression_*i*_ represents the normalized expression value of the *i*-th shared CSRDEG in each sample. The coefficient quantitatively reflects both the strength and direction (positive or negative) of association between each CSRDEG and disease status (CD vs. Control). Larger absolute coefficient values indicate greater contribution to the risk model.)

Common CSRDEGs present in all three models (Logistic-LASSO, SVM and RF) were identified using Venn analysis. The final diagnostic model and corresponding risk scores were computed by applying the LASSO-derived coefficients of these common CSRDEGs to the Combined dataset expression data. A Nomogram was generated using R packages to visualize individual gene contributions to the diagnostic model. Finally, a Decision Curve Analysis (DCA) plot created with the ggDCA package evaluated the clinical utility and net benefit of the CSRDEG diagnostic model.

### Calculation of cell senescence phenotype score and identification of disease subtypes based on combined dataset

To identify the potential mechanism, related biological features and pathways of CSRDEGs in CD, the single-sample gene-set enrichment analysis (ssGSEA) algorithm was used to calculate the Cellular senescence score (CSscore) based on the expression matrix of each sample in the combined dataset with help of GSVA package of R software.

The consensus clustering method of ConsensusClusterPlus package of R software was used to identify different disease subtypes (cluster1/cluster2) based on the expression of CSRDEGs in the Combined dataset. The parameters of the analysis were as follows: maxK=8, reps=50, pItem=0.8, pFeature=1, clusterAlg=“km”, distance=“euclidean”. The group expression difference of CSRDEGs among samples of different disease subtypes in the Combined dataset was calculated by Wilcoxon rank sum test. P value < 0.05 was considered statistically significant.

### Assessment of immune cell infiltration and its correlation with CSRDEGs

The enrichment score of 28 immune cells was calculated by ssGSEA algorithm with help of R-GSVA package (version 1.46.0) of R software (39) and used to represent the relative abundance of each immune cell infiltration in each sample (40). The enrichment score of 22 immune cells was calculated by CIBERSORT (41). The difference of infiltration levels of immune cells between different groups of the Combined dataset (CD group and controls, high score and low score groups, consistency cluster between different clusters) was shown by boxplot. The correlation between the immune cells of the combined dataset was visualized by correlation heat maps. The correlation between immune cells and genes of the Combined dataset was visualized by the correlation dot plot drawn by R package ggplot2.

### Statistical analysis

All data processing and analysis were performed using R software (Version 4.1.2). For comparisons between two groups of continuous variables, the independent Student’s t-test was employed to assess statistical significance for normally distributed variables, while the Mann-Whitney U test (i.e., Wilcoxon rank-sum test) was used to analyze differences between non-normally distributed variables. Unless otherwise specified, Pearson correlation analysis was applied to calculate correlation coefficients between different molecules. All statistical p-values were two-tailed, with p < 0.05 considered statistically significant.

## Results

### Identification and functional enrichment of cellular senescence-related differentially expressed genes in CD

A total of 281 CD patient tissues and 55 healthy control tissues were included (**Figure 1A**). After batch effect removal and normalization, a combined dataset was generated (**Supplementary Figure S1**). Differential expression analysis revealed 2,486 significantly upregulated genes and 2,105 downregulated genes in CD (**Figure 1B**). GSEA demonstrated significant enrichment of CD/Control genes in pathways, including PID_IL23_PATHWAY, PID_IL12_2PATHWAY, PID_IL6_7_PATHWAY, WP_INFLAMMATORY_BOWEL_DISEASE_SIGNALING, WP_INFLAMMATORY_RESPONSE_PATHWAY, WP_IL18_SIGNALING_PATHWAY (**Figure 1C**, **Supplementary Figure S2**, **Supplementary Table S3**). Intersection of 4,591 DEGs with 55 CSRGs identified 19 CSRDEGs (**Figure 1D**), including *CDKN1A*, *CDKN2A*, *CDKN2B*, *IGFBP7*, *IL1A*, *IL6*, *LMNA*, *MAPK3*, *NUAK1*, *PML*, *PTEN*, *RB1*, *SIRT1*, *SIRT6*, STAT3, *TERF2*, *TERT*, *TP53*, and *WRN*. Group comparison plots showed 11 downregulated genes (*CDKN1A*, *CDKN2A*, *CDKN2B*, *MAPK3*, *PTEN*, *SIRT1*, *SIRT6*, *TERF2*, *TERT*, *TP53*, and *WRN*) and 8 upregulated genes (*IGFBP7*, *IL1A*, *IL6*, *LMNA*, *NUAK1*, *PML*, *RB1*, and *STAT3*) (**Figure 1D**). Heatmaps validated expression differences of the 19 CSRDEGs (**Figure 1D**), and chromosomal mapping illustrated their distribution (**Supplementary Figure S3**). GO and KEGG enrichment analyze revealed that those genes involved in several important biological processes, including cellular senescence, regulation of mitotic cell cycle, regulation of cytokine production in inflammatory response. Detailed analysis results are presented in **Figure 1E**, **Supplementary Figure S4**, **Supplementary Table S4**.

### Identifying key module genes and potential therapeutic targets in CD via WGCNA and multi-dimensional interaction networks

**Figure 2:**
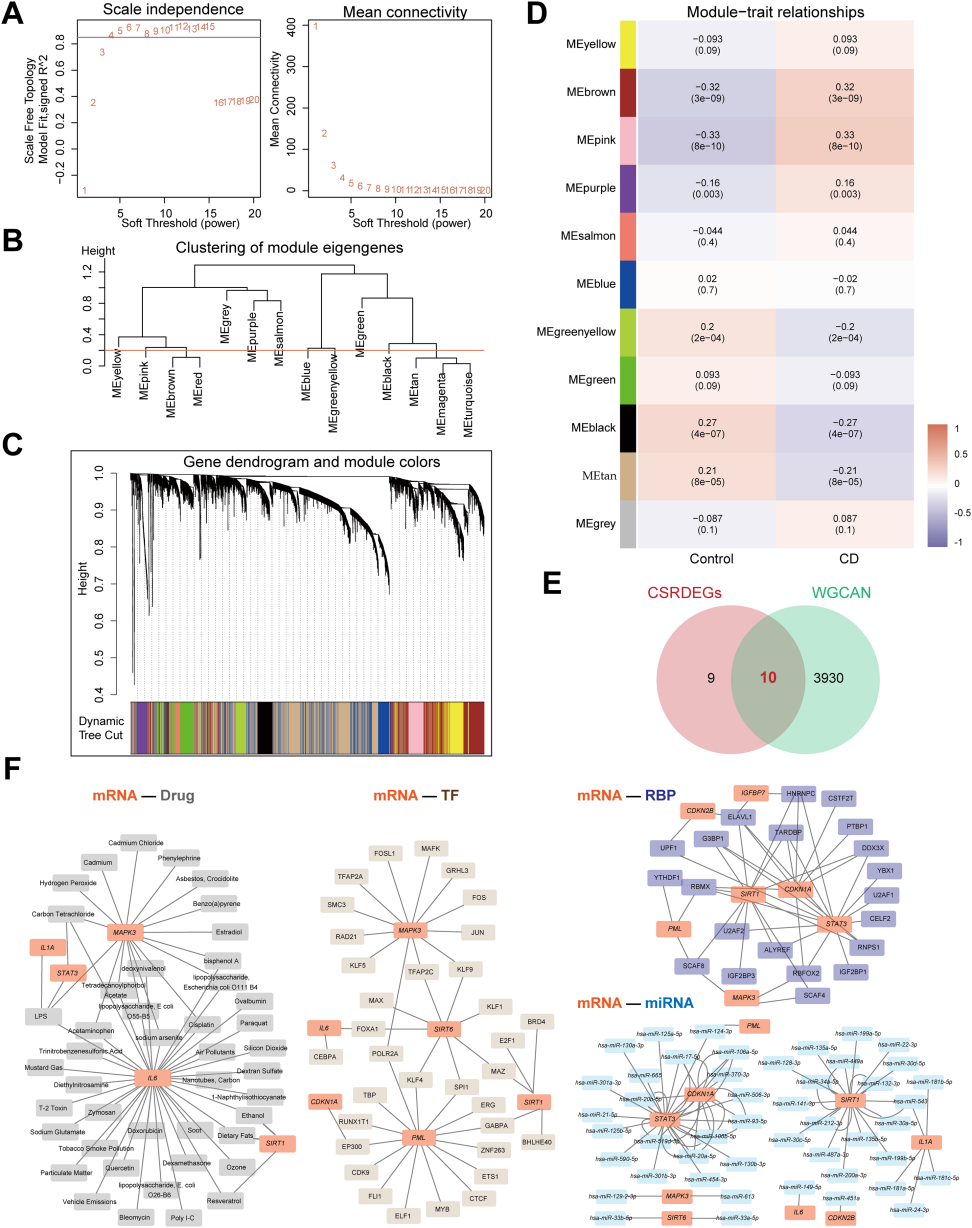
Identifying key module genes and potential therapeutic targets in CD via WGCNA and multi-dimensional interaction networks. **(A)** The selection of the value of the soft thresholding for a scale-free network of co-expression genes. **(B)** The show of the results of gene module aggregation. **(C)** Gene cluster dendrogram by dynamic tree cut algorithm. Genes are divided into various modules by hierarchical clustering, and different colors represent different modules, among which gray defaults to genes that cannot be classified into any module. **(D)** The heatmap of WGCNA (Weighted gene co-expression network analysis) analysis of key module genes. **(E)** The Venn diagram of the intersection of the CSRDEGs and WGCNA. 10 hub CSRDEGs (*CDKN1A*, *CDKN2B*, *IGFBP7*, *IL1A*, *IL6*, *MAPK3*, *PML*, *SIRT1*, *SIRT6*, and *STAT3*) were found. **(F)** The mRNA-Drug, mRNA-TF, mRNA-miRNA and mRNA-RBP regulatory network was constructed based on 10 hub CSRDEGs.

WGCNA was performed on the Combined dataset to identify co-expression modules. Initially, genes with top 40% variance were selected as input, and CD and Control groups were clustered using a cut height of 0.2. The optimal soft threshold power of 0.85 was determined (**Figure 2A**), and DEGs were clustered into 14 initial modules (e.g., MEyellow, MEpink, MEbrown; **Figures 2B, C**). These modules were further merged at a cut height of 0.2 (**Figure 2B**), resulting in 11 modules (e.g., MEyellow, MEbrown, MEpink) significantly associated with grouping (**Figures 2C, D**). After excluding the grey module (MEgrey), genes from 10 modules with significant correlations to grouping (P<0.05, |correlation|≥0.2) were intersected with CSRDEGs, yielding 10 module-phenotype genes (*CDKN1A*, *CDKN2B*, *IGFBP7*, *IL1A*, *IL6*, *MAPK3*, *PML*, *SIRT1*, *SIRT6*, and *STAT3*) (**Figure 2E**, **Supplementary Table S5**).

Four interaction networks were constructed based on these 10 genes (**Figure 2F**). mRNA-drug interaction network: Predicted via the CTD database (screening criteria: Reference Count>4), five mRNAs (*IL6*, *MAPK3*, *SIRT1*, *IL1A*, *STAT3*) interacted with 44 drugs (60 pairs; **Supplementary Table S6**). mRNA-TF interaction network: Screened using CHIPBase and hTFtarget databases (sample count>0), six genes (*CDKN1A*, *IL6*, *MAPK3*, *PML*, *SIRT1*, and *SIRT6*) interacted with 34 transcription factors (43 pairs; **Supplementary Table S7**). mRNA-miRNA interaction network: Identified via the miRDB database (≥5 website records), nine genes (*CDKN1A*, *CDKN2B*, *IL1A*, *IL6*, *MAPK3*, *PML*, *SIRT1*, *SIRT6*, and *STAT3*) interacted with 47 miRNAs (75 pairs; **Supplementary Table S8**). mRNA-RBP interaction network: Screened using the ENCORI database (clusterNum>5 and clipExpNum>5), 7 genes (*CDKN1A*, *CDKN2B*, *IGFBP7*, *MAPK3*, *PML*, *SIRT1*, *STAT3*) interacted with 21 RNA-binding proteins (47 pairs; **Supplementary Table S9**).

### Construction and validation of a multi-algorithm-integrated diagnostic model for CD

**Figure 3:**
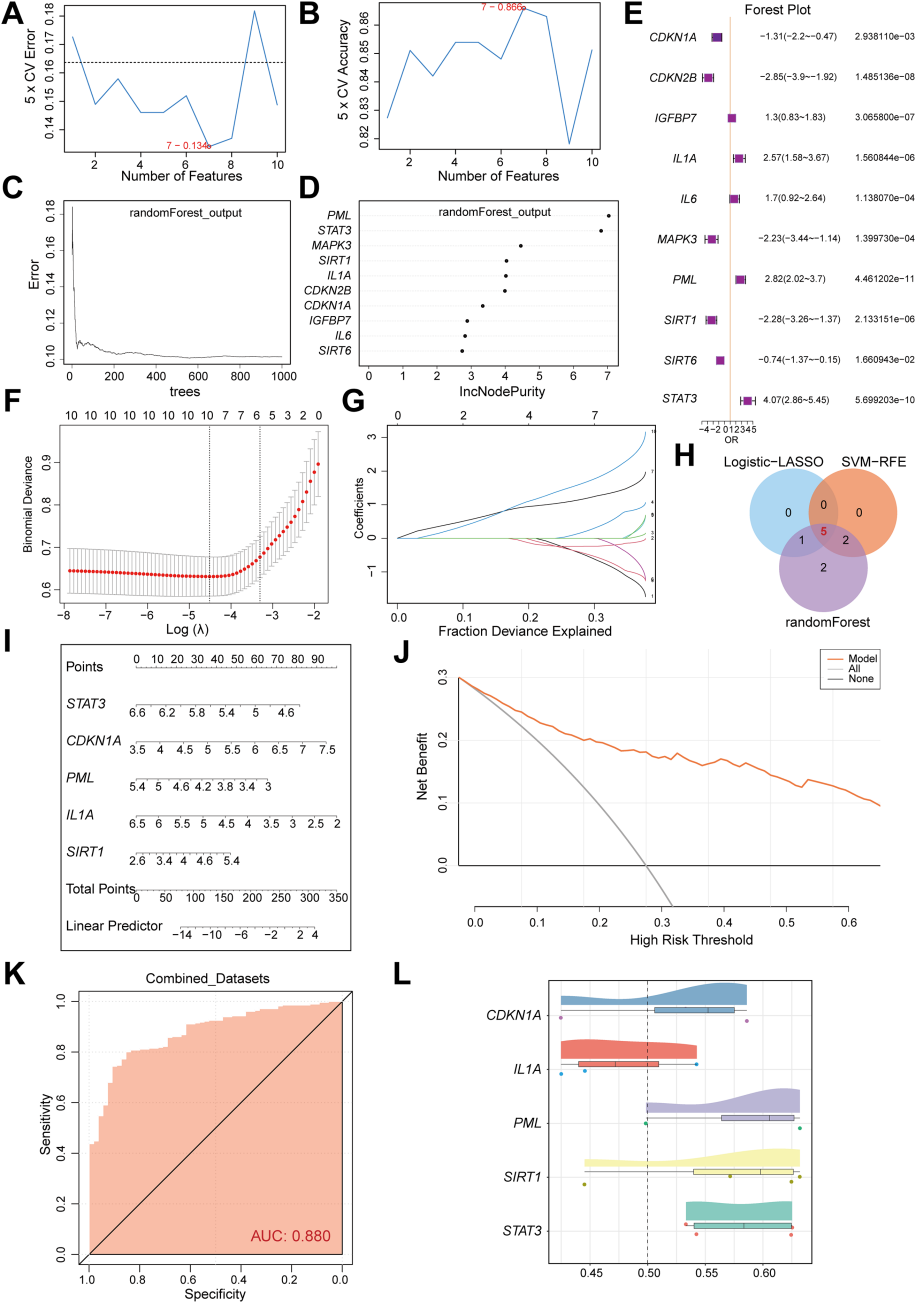
Construction and validation of a multi-algorithm-integrated diagnostic model for CD. **(A)** The number of genes with the lowest error rate is obtained by SVM algorithm. **(B)** The number of genes with the highest accuracy was obtained by SVM algorithm. **(C)** Model training error diagram of random forest algorithm. **(D)** Th show of CSRDEGs by random forest algorithm. **(E)** The forest plot of logistic regression model of CSRDEGs. **(F, G)** Identification of the minimum value and lambda value for diagnostic biomarker selection using the LASSO logistic regression algorithm. **(H)** The Venn diagram of the intersection of the Logistic-LASSO-CSRDEGs, SVM-RFE-CSRDEGs and Random Forest-CSRDEGs, and 5 CSRDEGs (*CDKN1A*, *IL1A*, *PML*, *SIRT1*, and *STAT3*) were found. **(I)** The nomogram of 5 CSRDEGs (*CDKN1A*, *IL1A*, *PML*, *SIRT1*, and *STAT3*) in the diagnosis of CD patients. **(J)** Decision curve analysis (DCA) of the nomogram model. **(K)** ROC curve of the CD diagnostic performance based on 5 CSRDEGs (*CDKN1A*, *IL1A*, *PML*, *SIRT1*, and *STAT3*). **(L)** Analysis of functional similarity between 5 CSRDEGs (*CDKN1A*, *IL1A*, *PML*, *SIRT1*, and *STAT3*).

A diagnostic model for CD was constructed based on 10 cellular senescence-related differentially expressed genes (CSRDEGs). The SVM model achieved the highest accuracy when selecting seven genes (**Figures 3A, B**). Random forest analysis (IncNodePurity>2) confirmed the diagnostic value of all 10 genes (**Figures 3C, D**). Logistic-LASSO regression further identified six key genes (CDKN1A, CDKN2B, IL1A, PML, SIRT1, STAT3) (**Figures 3E–G**), and intersection with SVM and random forest results yielded 5 consensus genes (*CDKN1A*, *IL1A*, *PML*, *SIRT1*, and *STAT3*) (**Figure 3H**). The diagnostic model was established using LASSO regression coefficients: RiskScore = *CDKN1A*×(-0.439) + *IL1A*×0.155 + *PML*×1.149 + *SIRT1*×(-0.413) + *STAT3*×1.609. A nomogram highlighted *IL1A* and *CDKN1A* as the primary contributors (**Figure 3I**). Decision curve analysis (DCA) and ROC curves validated the model’s clinical utility (AUC=0.880; **Figures 3J, K**). Functional similarity analysis (GOSemSim) indicated that PML exhibited the highest functional similarity to other genes (**Figure 3L**).

### Correlation analysis of immune infiltration features and cellular senescence genes in CD

**Figure 4:**
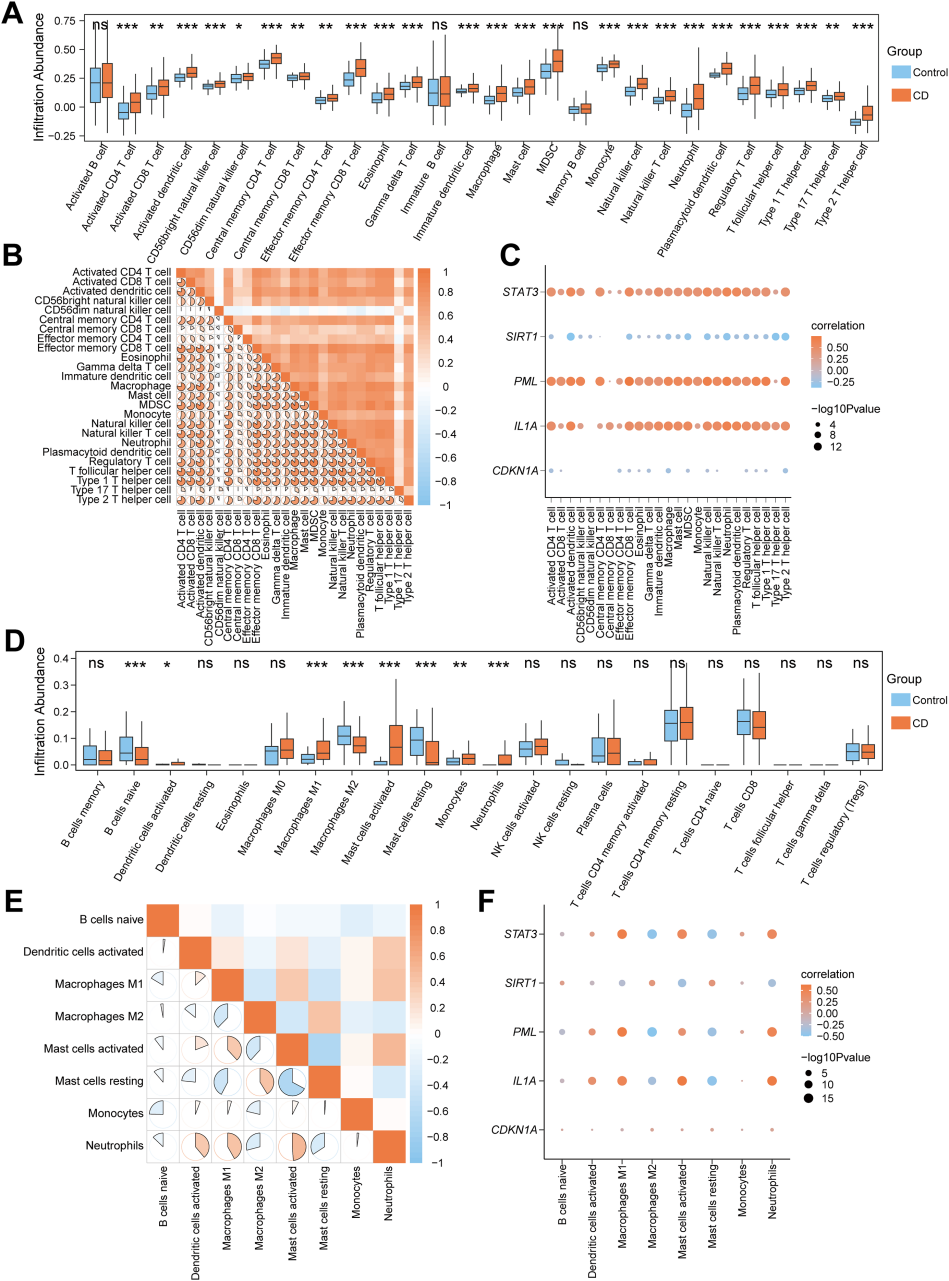
Correlation analysis of immune infiltration features and cellular senescence genes in CD. **(A)** Box plot for the enrichment score differences of 28 immune cells between CD and control group based on ssGSEA. **(B)** A heatmap showing the correlation of 25 differentially infiltrated immune cells. **(C)** Dot plot of correlation between 25 differentially infiltrated immune cells and 5 CSRDEGs (*CDKN1A*, *IL1A*, *PML*, *SIRT1*, and *STAT3*). **(D)** Box plot for the enrichment score differences of 22 immune cells between CD and control group based on CIBERSORT. **(E)** A heatmap showing the correlation of 8 differentially infiltrated immune cells. **(F)** Dot plot of correlation between 8 differentially infiltrated immune cells and 5 CSRDEGs (*CDKN1A*, *IL1A*, *PML*, *SIRT1*, and *STAT3*). ns, no significant, *P < 0.05, **P < 0.01 and ***P < 0.001.

To analyze the correlation between immune infiltration features and cellular senescence genes in CD, we performed ssGSEA on the Combined dataset to compare the infiltration of 28 immune cells between CD and Control groups. The results revealed that 25 immune cells (e.g., Activated CD4 T cell, Macrophage, MDSC) exhibited significant differences in abundance (P<0.05) (**Figure 4A**). Pearson correlation analysis demonstrated predominantly positive correlations among these 25 immune cells, with the strongest association observed between Macrophage and MDSC (**Figure 4B**). Further analysis of the correlations between five common CSRDEGs (*CDKN1A*, *IL1A*, *PML*, *SIRT1*, and *STAT3*) and immune cells (P<0.05) indicated that *STAT3*, *PML*, and *IL1A* were predominantly positively correlated with immune cells, whereas *SIRT1* and *CDKN1A* showed negative correlations. Notably, Effector memory CD8 T cells exhibited the strongest correlation with PML (**Figure 4C**). CIBERSORT analysis of 22 immune cells identified eight cells (e.g., B cells naïve, Macrophages M1/M2, Neutrophils) with significant abundance differences between CD and Control groups (**Figure 4D**). Pearson correlation analysis revealed balanced proportions of positive and negative correlations among these eight immune cells, with the strongest association observed between Mast cells activated and rested (**Figure 4E**). The correlation analysis between the 8 immune cells and 5 common CSRDEGs demonstrated that Mast cells activated showed the strongest correlation with *IL1A* (**Figure 4F**).

### Cellular senescence score grouping reveals heterogeneity of signature gene expression

**Figure 5:**
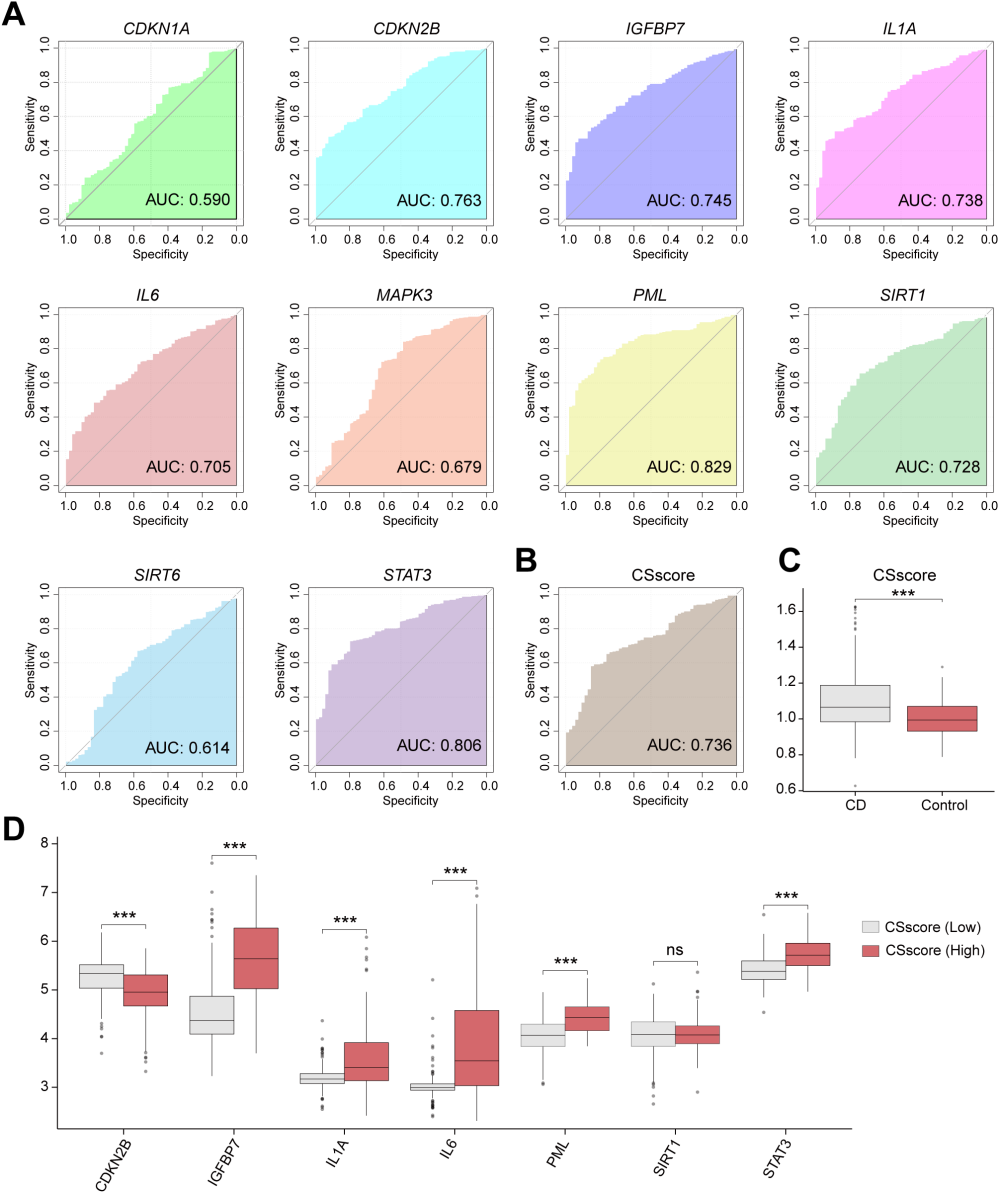
Cellular senescence score grouping reveals heterogeneity of signature gene expression. **(A)** ROC curve of the CD diagnostic performance based on different CSRDEGs (*CDKN1A*, *CDKN2B*, *IGFBP7*, *IL1A*, *IL6*, *MAPK3*, *PML*, *SIRT1*, *SIRT6*, and *STAT3*). **(B)** ROC curve of the CD diagnostic performance based on CSscore. **(C)** The CSscore difference between the CD and Control was compared. **(D)** Differential expression of 7 CSRDEGs (*CDKN2B*, *IGFBP7*, *IL1A*, *IL6*, *PML*, *SIRT1*, and *STAT3*) in CSscore (Low) and CSscore (High). ns, no significant, ***P < 0.001.

Based on the expression of 10 CSRDEGs (*CDKN1A*, *CDKN2B*, *IGFBP7*, *IL1A*, *IL6*, *MAPK3*, *PML*, *SIRT1*, *SIRT6*, and *STAT3*) in the combined dataset, we plotted diagnostic ROC curves, with 7 CSRDEGs demonstrating moderate diagnostic accuracy for CD (**Figure 5A**). These included *CDKN2B* (AUC=0.763), *IGFBP7* (AUC=0.745), *IL1A* (AUC=0.738), *IL6* (AUC=0.705), *PML* (AUC=0.829), *SIRT1* (AUC=0.728), and *STAT3* (AUC=0.806). Using the expression profiles of these seven CSRDEGs, we calculated the cellular senescence score (CSscore) for each sample via the ssGSEA algorithm. A diagnostic ROC curve was generated to evaluate the predictive capability of CSscore (**Figure 5B**), which showed moderate accuracy for CD diagnosis. Comparative analysis of CSscore between CD and control groups in the Combined dataset revealed statistically significant differences (**Figure 5C**). Samples were divided into High/Low CSscore groups based on the median CSscore. Group comparison plots demonstrated differential expression of the seven CSRDEGs between these groups (**Figure 5D**). All six CSRDEGs except SIRT1 exhibited significant expression differences between High and Low CSscore groups.

### Heterogeneity analysis of immune features based on CSscore grouping

**Figure 6:**
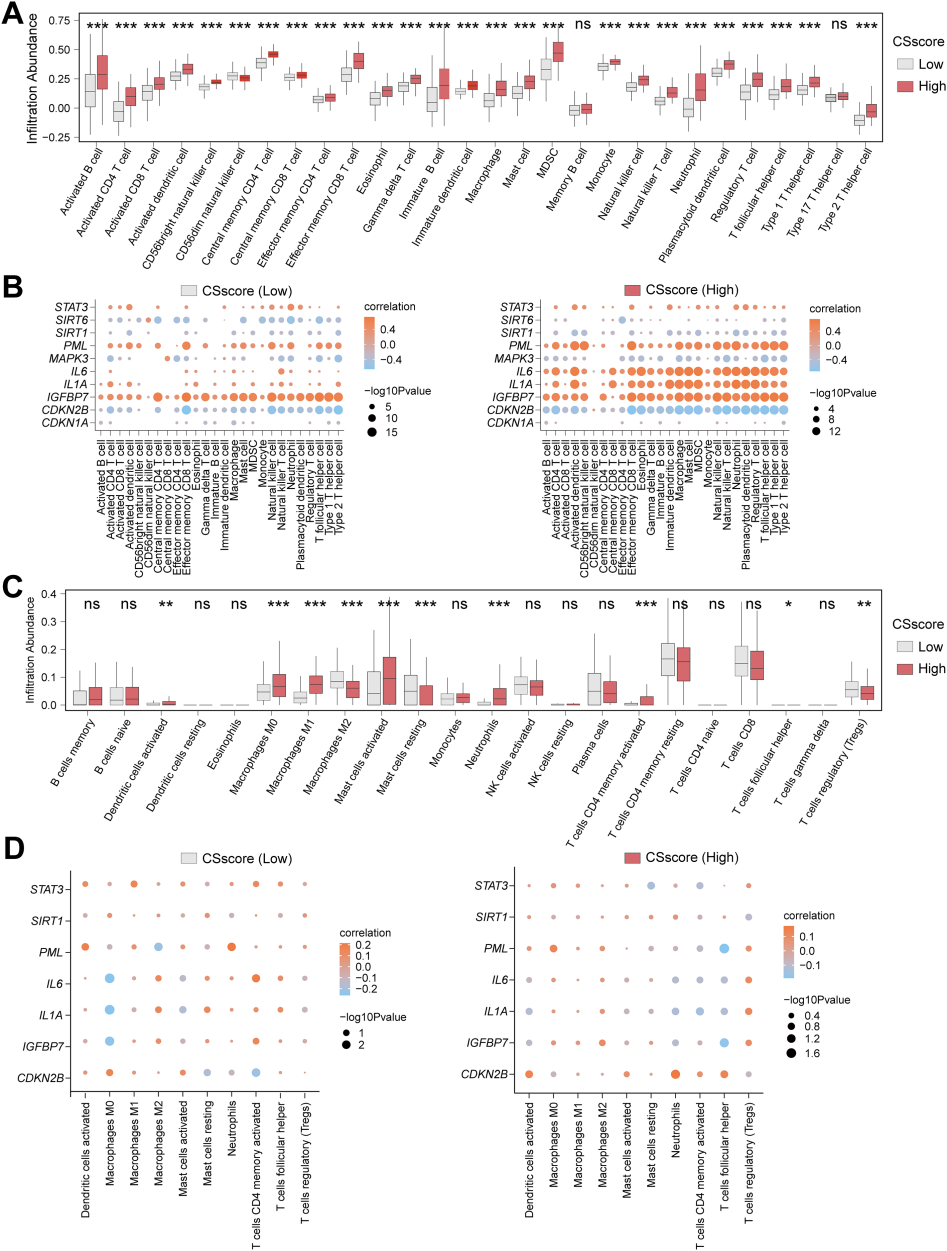
Heterogeneity analysis of immune features based on CSscore grouping. **(A)** Box plot for the enrichment score differences of 28 immune cells between CSscore (Low) and CSscore (High) group based on ssGSEA. **(B)** Dot plot of correlation between 26 differentially infiltrated immune cells and 7 CSRDEGs (*CDKN2B*, *IGFBP7*, *IL1A*, *IL6*, *PML*, *SIRT1*, and *STAT3*) in CSscore (Low) and CSscore (High) group. **(C)** Box plot for the enrichment score differences of 22 immune cells between CSscore (Low) and CSscore (High) group based on CIBERSORT. **(D)** Dot plot of correlation between 10 differentially infiltrated immune cells and 7 CSRDEGs (*CDKN2B*, *IGFBP7*, *IL1A*, *IL6*, *PML*, *SIRT1*, and *STAT3*) in CSscore (Low) and CSscore (High) group. ns, no significant, *P < 0.05, **P < 0.01 and ***P < 0.001.

To explore the immune feature differences between High and Low CSscore groups, we systematically analyzed CD samples in the Combined dataset using ssGSEA and CIBERSORT algorithms. Samples were divided into High/Low CSscore groups based on the median CSscore. ssGSEA results demonstrated that 26 out of 28 immune cells (e.g., activated T cells and macrophages) exhibited significantly different infiltration abundances between groups (**Figure 6A**). Pearson correlation analysis revealed that the 26 immune cells in the Low CSscore group were predominantly positively correlated, with Immature B cells and Activated B cells showing the strongest association (**Supplementary Figure S5A**). A similar positive correlation trend was observed in the High CSscore group, where Immature B cells and Activated B cells remained the most strongly correlated (**Supplementary Figure S5B**). Further analysis of the associations between immune cells and the seven CSRDEGs identified that in the Low CSscore group, Effector memory CD8 T cells showed the strongest correlation with *IGFBP7*, while *STAT3*, *PML*, and *IGFBP7* were primarily positively correlated with immune cells, and *CDKN2B* was predominantly negatively correlated (**Figure 6B**). In the High CSscore group, neutrophils exhibited the strongest correlation with *IL1A*, with *STAT3*, *PML*, and *IL6* showing positive correlations and *SIRT1* and *CDKN2B* showing negative correlations (**Figure 6B**).

To validate the robustness of these findings, CIBERSORT analysis of 22 immune cells identified 10 cells (e.g., M0/M1/M2 macrophages and regulatory T cells) with significantly different infiltration abundances between groups (P<0.05) (**Figure 6C**). Correlation analysis indicated balanced proportions of positive and negative correlations among the 10 immune cells in both High and Low CSscore groups, with resting and activated mast cells displaying the most significant association (**Supplementary Figures S5C, D**). Additionally, M0 macrophages showed the strongest correlation with *IL1A* in the Low CSscore group, while T follicular helper cells exhibited the strongest correlation with PML in the High CSscore group (**Figure 6D**). These results suggest that CSscore grouping effectively reflects the heterogeneity of the immune microenvironment, and key CSRDEGs demonstrate coordinated regulatory patterns with specific immune cells.

### Molecular subtyping and characterization of CD based on CSRDEGs

**Figure 7:**
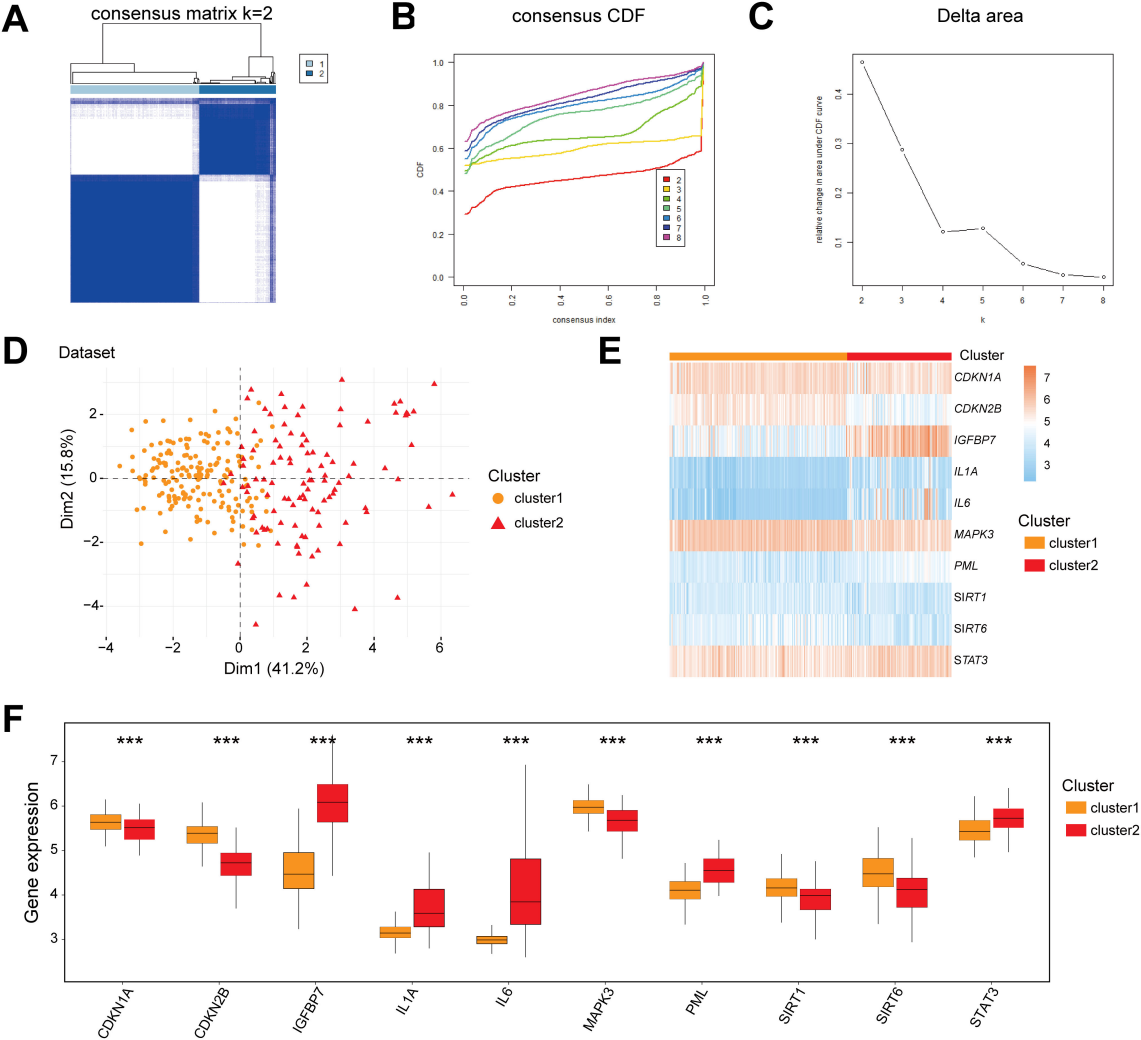
Molecular subtyping and characterization of CD based on CSRDEGs. **(A)** CD disease consistency cluster (K=2) result. **(B, C)** Consistent cluster cumulative Distribution function (CDF) diagram **(B)**, Area Delta diagram under CDF curve **(C)**. **(D)** PCA analysis results of two CD disease subtypes (cluster1 and cluster2) were presented. **(E)** The heatmap of 10 hub CSRDEGs (*CDKN1A*, *CDKN2B*, *IGFBP7*, *IL1A*, *IL6*, *MAPK3*, *PML*, *SIRT1*, *SIRT6*, and *STAT3*) in different CD disease subtypes. **(F)** The box plot of 10 hub CSRDEGs (*CDKN1A*, *CDKN2B*, *IGFBP7*, *IL1A*, *IL6*, *MAPK3*, *PML*, *SIRT1*, *SIRT6*, and *STAT3*) in different CD disease subtypes. ***P < 0.001.

To decipher the molecular heterogeneity of CD in the Combined dataset, this study classified CD samples into disease subtypes using consensus clustering based on the expression profiles of 10 CSRDEGs. The clustering results demonstrated that the stability was optimal when the cluster number k=2, as evidenced by the consensus cumulative distribution function (CDF) curves and Delta area values (**Figures 7A–C**). Cluster1 contained 176 samples, while cluster2 included 105 samples. Principal component analysis (PCA) further confirmed significant transcriptomic differences between the two subtypes (**Figure 7D**). Heatmap visualization revealed marked heterogeneity in the expression of the 10 CSRDEGs between cluster1 and cluster2 (**Figure 7E**). Mann-Whitney U tests indicated that all genes exhibited statistically significant expression differences between the subtypes (P<0.05), with *CDKN1A*, *CDKN2B*, *MAPK3*, *SIRT1*, and *SIRT6* significantly upregulated in cluster1, whereas *IGFBP7*, *IL1A*, *IL6*, *PML*, and *STAT3* were highly expressed in cluster2 (**Figure 7F**). These findings suggest that molecular subtyping based on CSRDEGs effectively distinguishes disease heterogeneity in CD, with distinct subtypes potentially corresponding to specific gene regulatory patterns.

### Immune infiltration heterogeneity and gene interaction networks across CD subtypes

**Figure 8:**
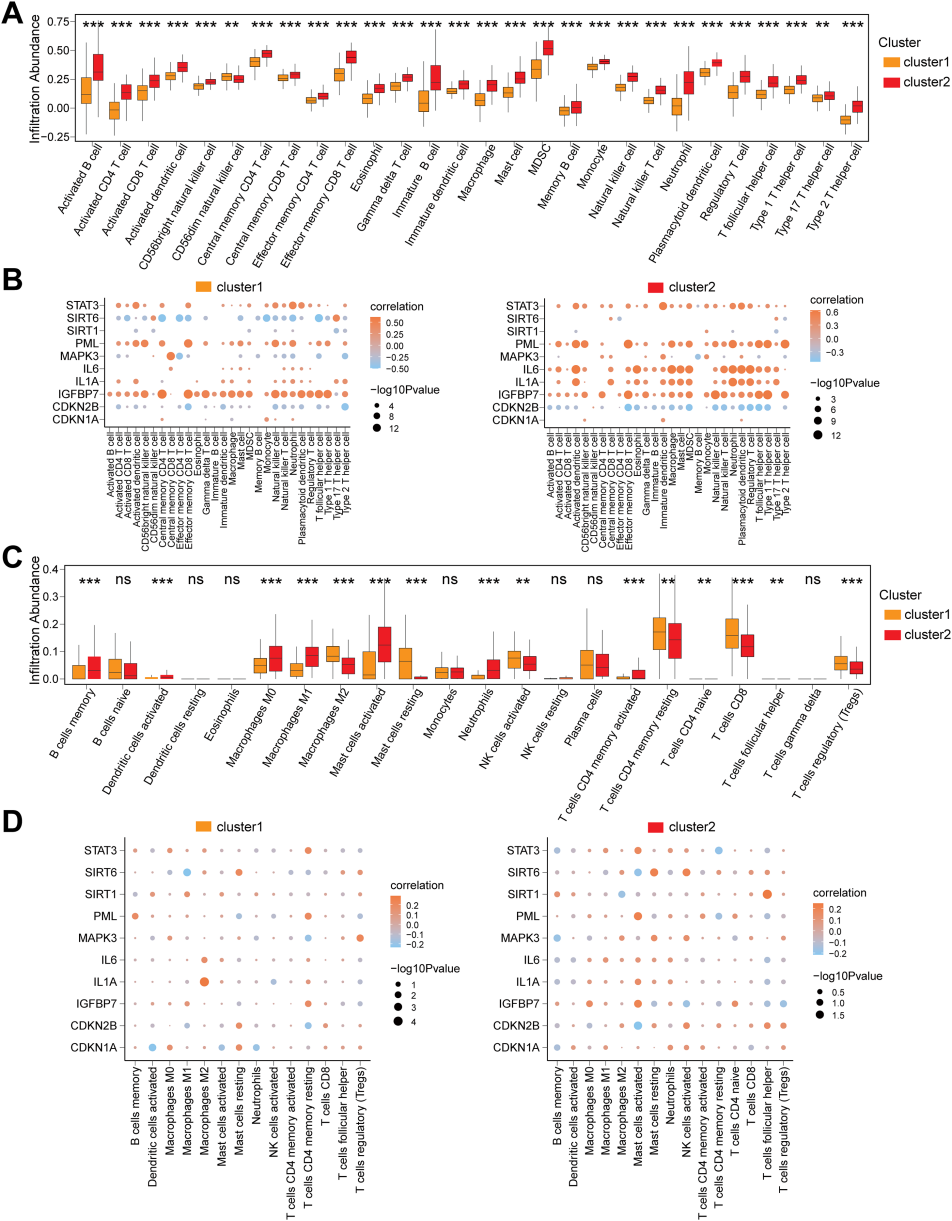
Immune infiltration heterogeneity and gene interaction networks across CD Subtypes. **(A)** Box plot for the enrichment score differences of 28 immune cells between cluster 1 and cluster 2 group based on ssGSEA. **(B)** Dot plot of correlation between 28 differentially infiltrated immune cells and 10 CSRDEGs (*CDKN1A*, *CDKN2B*, *IGFBP7*, *IL1A*, *IL6*, *MAPK3*, *PML*, *SIRT1*, *SIRT6*, and *STAT3*) in cluster 1 and cluster 2 group. **(C)** Box plot for the enrichment score differences of 22 immune cells between cluster 1 and cluster 2 group based on CIBERSORT. **(D)** Dot plot of correlation between 15 differentially infiltrated immune cells and 10 CSRDEGs (*CDKN1A*, *CDKN2B*, *IGFBP7*, *IL1A*, *IL6*, *MAPK3*, *PML*, *SIRT1*, *SIRT6*, and *STAT3*) in cluster 1 and cluster 2 group. ns, no significant, **P < 0.01 and ***P < 0.001.

To investigate the immune feature differences between CD subtypes (cluster1/cluster2) in the Combined dataset, we conducted a systematic analysis using ssGSEA and CIBERSORT algorithms. The ssGSEA results demonstrated that the infiltration abundances of 28 immune cells (e.g., Activated B cell, Macrophage, Neutrophil) were significantly different between cluster1 and cluster2 (P<0.05) (**Figure 8A**). Pearson correlation analysis revealed that the 28 immune cells exhibited widespread positive correlations in both subtypes, with the strongest correlation observed between Immature B cells and Activated B cells (**Supplementary Figures S6A, B**). Further analysis of the associations between immune cells and the 10 CSRDEGs identified that in cluster1, Central memory CD4 T cells showed the strongest correlation with *IGFBP7*, while *STAT3*, *PML*, and *IGFBP7* were primarily positively correlated with immune cells, and CDKN2B was predominantly negatively correlated (**Figure 8B**). In cluster2, Type 1 T helper cells exhibited the strongest correlation with IGFBP7, with STAT3, PML, and IL6 showing positive correlations and CDKN2B remaining negatively correlated (**Figure 8B**).

Validation using the CIBERSORT algorithm identified 15 out of 22 immune cells (e.g., Macrophages M0/M1/M2, T cells regulatory (Tregs)) with significantly different infiltration abundances between subtypes (P<0.05) (**Figure 8C**). Correlation analysis indicated that in cluster1, the infiltration abundance of T cells CD4 naïve was entirely zero, and the remaining 14 immune cells showed balanced proportions of positive and negative correlations, with the strongest association observed between Mast cells resting and activated (**Supplementary Figure S6C**). In cluster2, the 15 immune cells displayed similar trends in positive and negative correlations, and Mast cells resting-activated remained the most strongly correlated (**Supplementary Figure S6D**). Gene-immune interaction analysis demonstrated that in cluster1, Macrophages M2 showed the strongest correlation with *IL1A* (**Figure 8D**), while in cluster2, T cells follicular helper exhibited the most significant association with *SIRT1* (**Figure 8D**).

## Discussion

CD is a multifaceted IBD that is traditionally viewed through the lens of immune dysregulation (42). This study systematically revealed the core regulatory role of CSRDEGs in CD by integrating multi-cohort gene expression data from the public database. Through differential expression analysis, WGCNA network construction, and a multi-algorithm integration model, we identified 10 CSRDEGs. Notably, pro-inflammatory and pro-senescence genes including *STAT3*, *IL6*, and *IL1A* showed significant upregulation, while anti-senescence genes such as *SIRT1* and *CDKN1A* were markedly downregulated. This dysregulation suggests that cell cycle exit and senescence signaling pathways are fundamentally altered in CD. Remarkably, a multi-algorithm diagnostic model constructed based on CSRDEGs not only demonstrated excellent clinical discriminative capability, but its molecular subtype classification (cluster1/cluster2) also revealed heterogeneity in CD, providing new perspectives for precise subtyping. Further ssGSEA and CIBERSORT analyses revealed that macrophage and T cells regulatory enrichment in the high CSscore group showed strong positive correlation with *STAT3*/*PML*, suggesting that the synergistic effect between cellular senescence and immune microenvironment remodeling may serve as a key mechanism driving CD progression. Our findings not only enrich current knowledge about CD but also offer potential therapeutic targets that could be exploited.

The CSRDEGs identified in this study exhibit well-defined biological functions in cellular senescence, inflammatory, and fibrotic pathways. For instance, STAT3, a central node in JAK-STAT signaling (43), plays a pivotal role in NOD2 risk allele-driven CD pathogenesis (44). The seminal work by Nayar S et al. (44) demonstrated that NOD2 loss-of-function remodels the macrophage-fibroblast interaction network, activates STAT3-dependent signaling, and subsequently regulates the expression of pro-fibrotic factors (e.g., IL-11 and WT1) in fibroblasts along with inflammatory mediators (e.g., IL-6 and CXCL13) in macrophages. It is noteworthy that the high expression of STAT3 in the cluster2 subtype in this study may suggest that this subtype has the potential for intestinal fibrosis. Furthermore, IL-6 activates the JAK1/STAT3 signaling axis via gp130 receptor engagement, inducing HIF1A mRNA transcription and stabilizing HIF1α protein. The HIF1α-ERRα interaction suppresses ERRα transcriptional activity, with reduced function of this critical mitochondrial biogenesis regulator exacerbating mitochondrial dysfunction and promoting inflammaging (45). Crucially, STAT3 directly binds the HIF1A promoter to enhance its expression. Simultaneously, IL-6-mediated ERRα inhibition potentially establishes a self-sustaining regulatory loop through PGC1α downregulation. This cascade amplifies local inflammatory signals while shifting cellular metabolism toward glycolysis via suppressed oxidative phosphorylation capacity, thereby establishing a proinflammatory-metabolic dysregulation feedback loop in chronic inflammation (45). Such mechanisms demonstrate significant complementarity with IL-18 pathway dysregulation observed in the S2FCD subtype proposed by Tindle et al. (12).

Of note, SIRT1, an NAD+-dependent class III histone deacetylase, exhibits diminished expression that compromises cellular defenses against oxidative stress. Studies indicate that SIRT1 maintains redox homeostasis by activating FOXO3-dependent antioxidant genes (e.g., catalase, MnSOD) and deacetylating nuclear factor erythroid 2-related factor 2 (Nrf2) (46). Its downregulation may impair antioxidant capacity in intestinal epithelial cells, aligning with observed accumulations of reactive oxygen species (ROS)-related damage markers in CD patient-derived organoid cultures (PDOs), particularly in the S2FCD subtype (12). Importantly, SIRT1 suppresses NF-κB transcriptional activity by specifically deacetylating the Lys310 residue of NF-κB p65 (47). In CD’s chronic inflammatory microenvironment, reduced SIRT1 expression may attenuate NF-κB pathway inhibition, leading to excessive secretion of pro-inflammatory cytokines (e.g., IL-6, IL-8) and matrix metalloproteinases (e.g., MMP9)—core components of the senescence-associated secretory phenotype (SASP) (46). Additionally, promyelocytic leukemia protein (PML) organizes into highly ordered PML nuclear bodies (NBs) within the nucleus. These supramolecular complexes and membrane-less subcellular organelles critically regulate DNA damage response, apoptosis, and cellular senescence pathways (23, 48, 49). Research reveals that functional loss of SP140, an epigenetic “reader” and essential PML-NB component, drives CD pathogenesis through dysregulated topoisomerase activity in macrophages (50). Coupled with this study’s findings of strong PML-immune cell correlations and PML’s role as a key CD subtype classifier, these observations suggest that PML may critically influence inflammatory progression in CD, though its precise mechanisms require further investigation.

Machine learning algorithms have significantly promoted the construction of clinical models. This study integrated multiple machines learning algorithms, including Support SVM, RF, and LASSO, to construct a CD diagnostic model based on five CSRDEGs (*CDKN1A*, *IL1A*, *PML*, *SIRT1*, and *STAT3*), with robust diagnostic performance validated. Nomogram analysis revealed that IL1A and CDKN1A contributed most significantly to the model, suggesting their potential roles as key diagnostic drivers. Decision curve analysis demonstrated high net clinical benefit, particularly across broad risk threshold ranges, outperforming “all-positive” or “all-negative” diagnostic strategies. OC curve analysis further confirmed the model’s diagnostic accuracy, highlighting its superior sensitivity and specificity in distinguishing CD patients from healthy controls. These findings collectively support the clinical utility of the five CSRDEGs as potential biomarkers. Functional similarity analysis uncovered potential synergistic interactions among the five CSRDEGs across BP, MF, and CC. Cloud-rain plot visualization indicated *PML* as the gene with the highest functional similarity to others, suggesting its central role in senescence-associated pathways. Notably, CDKN1A, IL1A, SIRT1, and STAT3 also exhibited substantial functional overlap, implying their coordinated involvement in regulating cellular senescence and inflammatory responses through shared signaling cascades. These insights provide a theoretical foundation for further exploration of these genes in CD pathogenesis and potential therapeutic targeting of senescence-related mechanisms. The CSRDEG-based diagnostic model offers a novel perspective and tool for CD diagnosis. Compared to existing methods, this model leverages senescence-associated genes, potentially enabling earlier detection of disease progression. Furthermore, the five CSRDEGs (*CDKN1A*, *IL1A*, *PML*, *SIRT1*, *STAT3*) are all well-characterized in senescence and inflammation, ensuring biological coherence and enhancing model interpretability. Future research should investigate their utility in therapeutic monitoring and prognostic evaluation, such as dynamically tracking gene expression to assess treatment efficacy or predict relapse risk.

CD is a disease characterized by immune response disorder, and in-depth analysis of it can help us understand the nature of the disease. This study comprehensively analyzed immune characteristics across disease control groups, high/low scoring subgroups, and CD subtypes in the combined dataset using CIBERSORT and ssGSEA algorithms. Results revealed significant differences in immune cell infiltration abundance between subgroups and subtypes, with distinct correlation patterns among immune cells and between immune cells and CSRDEGs, providing critical insights for therapeutic strategy development and immune mechanism exploration. Notably, IL1A (encoding IL-1α) exhibited significant correlations with neutrophil and macrophage subpopulation (M0/M1/M2) infiltration across all disease subgroups. IL-1α, constitutively expressed as an active pro-form in all cells, is uniquely localized to the nucleus, cytoplasm, and cell membrane with compartment-specific functions (51). Its underrecognized role in CD pathogenesis has been highlighted by studies demonstrating that damaged intestinal epithelial cells (IECs) release substantial IL-1α during spontaneous ileitis and DSS-induced colitis (52). Downregulated IL1A in TLR4-signaling pathways (53) further indicates its context-dependent immunomodulatory function, positioning it as a viable therapeutic target. Neutralizing antibodies (e.g., FLO1 mAb) could disrupt IL-1α-driven barrier dysfunction while promoting probiotic enrichment (52), as evidenced by reduced ileitis/colitis severity upon blockade. This cytokine subsequently induces inflammatory mediators (IL-1β, Cox2, MPO) and disrupts intestinal barrier integrity. Blocking IL-1α with FLO1 mAb (a murine-specific monoclonal antibody) markedly reduced ileitis/colitis severity, while paradoxically elevating IL-18 (a gut barrier integrity promoter) and inflammatory markers. IL-1α silencing was associated with microbial community restructuring toward probiotic enrichment, suggesting its dual role in modulating gut microbiome functionality and CD progression. Emerging evidence implicates IL-1α in pathogenic immune regulation: senescent immune systems produce “detrimental” IL-1α that mediates immunosuppression in lung cancer (54), while necroptotic cancer cells release IL-1α to recruit immunosuppressive neutrophils/macrophages, perpetuating tumor immunosuppression post-mortem (55). These findings underscore the necessity to dissect IL-1α’s multifaceted immunoregulatory roles in CD for developing novel therapeutic strategies.

This study reveals that CD subtypes (cluster1/cluster2) exhibit heterogeneity that likely corresponds to distinct clinical phenotypes and therapeutic requirements. Cluster1 is characterized by elevated CDKN1A/SIRT1 expression. High CDKN1A (p21) levels indicate enhanced cell cycle arrest (56), potentially marking a disease remission phase or low-inflammatory subtype. Experimental validation should include SIRT1 overexpression in CD patient-derived organoids to assess barrier function recovery. Therapeutically, these patients may benefit from SIRT1 agonists (e.g., SRT1720) to mitigate intestinal epithelial senescence by enhancing antioxidant capacity (46). Crucially, epigenetic modulation via miR-4262 inhibition, shown to upregulate SIRT1 and suppress inflammatory apoptosis in IBD (57) that represents a novel complementary strategy to restore redox homeostasis in this subtype. In contrast, cluster 2 demonstrates IL-6/STAT3 overexpression. Crucially, sustained activation of STAT3 has been mechanistically linked to the development of the fibrostenotic phenotype in CD (44). This pathological role positions STAT3 as a compelling therapeutic target for this patient subset. Supporting this notion, JAK inhibitors (e.g., upadacitinib), which act upstream by inhibiting the IL-6-gp130-STAT3 axis, have demonstrated clinical efficacy in improving endoscopic outcomes in CD patients (58). The role of STAT3 in intestinal inflammation is complex and context-dependent. Genetic studies have identified STAT3 as an IBD susceptibility locus in both pediatric and adult populations. Furthermore, the IL-10/STAT3 signaling axis plays a vital protective and anti-inflammatory role in the gut, essential for controlling inflammation and preventing tissue damage (59, 60). Mechanistically, IL-10 binding to its receptor complex (IL10RA/IL10RB) activates Jak1 and Tyk2 kinases, leading to STAT3 phosphorylation. Activated STAT3 then translocates to the nucleus to transcribe genes that suppress inflammation (61). This highlights a critical dichotomy: while dysregulated, persistent IL-6-driven STAT3 activation promotes fibrosis, the IL-10-driven STAT3 pathway is fundamentally anti-inflammatory. Based on our cluster 2 findings (IL-6/STAT3 overexpression linked to fibrosis) and the established pro-fibrotic role of sustained STAT3 activation, we propose that targeted STAT3 inhibition holds specific therapeutic promise for cluster 2 patients with fibrostenotic CD. Beyond conventional JAK inhibitors, clinically validated small-molecule inhibitors targeting PML, such as Arsenic Trioxide (used successfully in acute promyelocytic leukemia) (62), warrant investigation for CD-associated fibrosis. For translational validation of STAT3’s direct role in fibrosis within the cluster 2 context, we propose an *in vitro* experimental approach: Utilizing macrophage-fibroblast co-culture systems modeling the fibrotic niche, specific STAT3 knockdown (e.g., via siRNA or CRISPR-Cas9) should be performed. The subsequent quantification of key fibrotic markers (e.g., COL1A1, α-SMA (63)) would provide direct mechanistic evidence of STAT3’s contribution to the fibrotic phenotype observed in this cluster. These findings underscore the mechanistic heterogeneity underlying CD pathogenesis, emphasizing the need to elucidate subtype-specific molecular pathways to enable precision subtyping and advance tailored therapeutic strategies.

This study systematically elucidates the regulatory roles of CSRDEGs in CD, yet several limitations warrant attention. Firstly, Sample Heterogeneity and Confounding Factors. Our analysis relied on public databases encompassing unstratified intestinal biopsy sites (ileum vs. colon) and disease phases (active vs. remission). Although rigorous standardization and batch-effect correction were applied to minimize technical and biological variations, residual confounding effects from intrinsic tissue heterogeneity cannot be fully excluded. This is particularly relevant given CD’s patchy inflammation pattern (64), where adjacent mucosal zones may exhibit divergent molecular profiles. Such variability could impact the generalizability of gene expression signatures. Future studies should prioritize stratified sampling or subgroup analyses based on standardized anatomic and clinical annotations. Secondly, Mechanistic Validation Gap. Despite robust bioinformatic predictions (e.g., WGCNA, GSEA) and stable model performance, the causal roles of CSRDEGs in intestinal barrier dysfunction or fibrosis remain experimentally unvalidated. The absence of functional validation using pathophysiologically relevant models, such as CD patient-derived organoids (12) or *in vivo* systems—limits translational inference. We explicitly acknowledge this gap and plan targeted experiments (e.g., CRISPR-based gene editing in 3D organoid models) to verify the biological impact of prioritized CSRDEGs. Thirdly, Immune Microenvironment. While ssGSEA and CIBERSORT algorithms revealed significant shifts in immune cell abundance, bulk transcriptome resolution inherently precludes assessment of functional immune states (e.g., T-cell exhaustion, macrophage polarization). And the state of immune cells (especially the expression of MHCI/MHCII) remains a problem that we need to consider. This constraint may lead to incomplete characterization of immune senescence dynamics. Finally, Clinical Application Faces Challenges. The tissue-dependent nature of our diagnostic model restricts its utility for non-invasive applications. Developing blood-based biomarkers (e.g., SASP factors) or imaging-compatible signatures represents an unmet need for point-of-care deployment.

Building on this study, future research should prioritize the following directions. Firstly, longitudinal tracking of CD progression integrated with single-timepoint multi-omics data (transcriptomic, proteomic) (65) to elucidate dynamic interactions between cellular senescence and immune responses. Secondly, leveraging single-cell RNA sequencing (scRNA-seq) and spatial transcriptomics (66) to map cell type-specific CSRDEG expression patterns in intestinal epithelial cells, fibroblasts, Treg cells, and other subpopulations, clarifying their compartmentalized functions. Thirdly, given the established role of specific pathogens (e.g., AIEC (67), MAP (68)) in CD pathogenesis, it is imperative to investigate how CSRDEGs modulate host-pathogen interactions (69). We will focus on exploring the following aspects in the future. (1) SASP-mediated alterations in epithelial barrier integrity affecting pathogen adhesion/invasion (e.g., AIEC). (2) senescence-associated immune dysregulation (e.g., impaired macrophage phagocytosis or defective antigen presentation) facilitating intracellular pathogen persistence (e.g., MAP). (3) CSRDEG-driven changes in autophagy, inflammasome activation, or antimicrobial peptide production that may reshape the microbial niche. Integrating host transcriptomics with metagenomic sequencing and targeted pathogen detection in future cohorts will be essential to decode these relationships. (4) Intriguingly, our differential expression analysis revealed significant upregulation of MHC-II genes (e.g., HLA-DMA, HLA-DPA1, HLA-DPB1; see **Supplementary Table S10**). Although current data limitations preclude direct correlation analysis between CSRDEGs and MHC expression, future studies should systematically investigate how senescence-associated genes modulate antigen-presenting machinery. (5) Analyzing associations between immunosenescence markers (e.g., CD28^-^CD57^+^ T cells (70), SA-β-gal^+^ macrophages (71)) and CSRDEGs in elderly CD cohorts (>60 years) may uncover age-specific vulnerabilities to pathogen-driven inflammation, guiding the development of age-tailored therapeutic strategies. Notably, while the current study utilized publicly available datasets lacking detailed microbial metadata, limiting direct correlation analysis between CSRDEGs and pathogen burden, resolving this mechanistic gap represents a critical frontier for understanding CD heterogeneity.

## Conclusion

In summary, this study systematically unveils the central regulatory roles of CSRDEGs in CD heterogeneity and immune microenvironment imbalance through multidimensional analyses. It preliminarily explores the complex interplay between cellular senescence and immune dysregulation, providing a groundbreaking perspective for understanding CD pathophysiology. The CSRDEG-based diagnostic model and molecular subtyping (cluster1/cluster2) offer innovative tools for precision diagnosis and treatment, while the identification of key targets (e.g., IL1A, STAT3, PML) lays a foundation for developing senotherapeutics targeting senescence pathways, such as SASP inhibitors and JAK/STAT3 blockers. Collaborative efforts across disciplines will be essential to validate these findings, optimize targeted interventions, and ultimately improve the quality of life for patients with CD and other chronic inflammatory diseases.

## Data Availability

The datasets presented in this study can be found in online repositories. The names of the repository/repositories and accession number(s) can be found in the article/**Supplementary Material**.

## Glossary

CD: Crohn’s disease. IBD, inflammatory bowel disease
WHO: World Health Organization
SASP: senescence-associated secretory phenotype
PDOs: patient-derived organoids
IDICD: immune-deficient infectious subtype
S2FCD: senescence-driven fibrostenotic subtype
TEER: transepithelial electrical resistance
CSRGs: Cellular senescence related genes
DEGs: differential expression genes
CSRDEGs: cellular senescence related differentially expressed genes
GSEA: Gene Set Enrichment Analysis
ssGSEA: single-sample gene-set enrichment analysis
WGCNA: Weighted Gene Co-expression Network Analysis
GO: Gene Ontology
KEGG: Kyoto Encyclopedia of Genes and Genomes
FDR: false discover rate
FC: fold change
CTD: Comparative Toxicogenomics Database
TF: transcription factors
RBP: RNA-binding proteins
LASSO: Least Absolute Shrinkage and Selection Operator
SVM: Support Vector Machine
RF: random forest
DCA: Decision Curve Analysis
CSscore: Cellular senescence score
BP: Biological Processes
CC: Cellular Components
ROS: reactive oxygen species
PML: promyelocytic leukemia protein
IECs: intestinal epithelial cells
scRNA-seq: single-cell RNA sequencing

## References

[1] Gordon H Burisch J Ellul P Karmiris K Katsanos K Allocca M. ECCO guidelines on extraintestinal manifestations in inflammatory bowel disease. J Crohn’s colitis. (2024) 18:1–37. doi: 10.1093/ecco-jcc/jjad108 37351850

[2] Loy L Roda G Fiorino G Allocca M Furfaro F Argollo M. Detection and management of early stage inflammatory bowel disease: an update for clinicians. Expert Rev Gastroenterol Hepatol. (2019) 13:547–55. doi: 10.1080/17474124.2019.1605291 31007098

[3] Rogler G Singh A Kavanaugh A Rubin DT. Extraintestinal manifestations of inflammatory bowel disease: current concepts, treatment, and implications for disease management. Gastroenterology. (2021) 161:1118–32. doi: 10.1053/j.gastro.2021.07.042 PMC8564770 34358489

[4] Xu L He B Sun Y Li J Shen P Hu L. Incidence of inflammatory bowel disease in urban China: A nationwide population-based study. Clin Gastroenterol Hepatol. (2023) 21:3379–86.e29. doi: 10.1016/j.cgh.2023.08.013 37660767

[5] Wylezinski LS Gray JD Polk JB Harmata AJ Spurlock CF 3rd. Illuminating an invisible epidemic: A systemic review of the clinical and economic benefits of early diagnosis and treatment in inflammatory disease and related syndromes. J Clin Med. (2019) 8. doi: 10.3390/jcm8040493 PMC6518102 30979036

[6] Adamina M Minozzi S Warusavitarne J Buskens C Chaparro M Verstockt B. ECCO guidelines on therapeutics in Crohn’s disease: surgical treatment. J Crohn’s colitis. (2024) 18(10):1556-82. doi: 10.1093/ecco-jcc/jjae089 38878002

[7] Gonczi L Lakatos L Kurti Z Golovics PA Pandur T David G. Incidence, prevalence, disease course, and treatment strategy of Crohn’s disease patients from the Veszprem cohort, western Hungary: A population-based inception cohort study between 2007 and 2018. J Crohn’s colitis. (2023) 17:240–8. doi: 10.1093/ecco-jcc/jjac132 36087109

[8] Shehab M Alrashed F Heron V Restellini S Bessissow T. Comparative efficacy of biologic therapies for inducing response and remission in Fistulizing Crohn’s disease: systematic review and network meta-analysis of randomized controlled trials. Inflamm Bowel Dis. (2023) 29:367–75. doi: 10.1093/ibd/izac103 35604382

[9] Li X Li C Zhang W Wang Y Qian P Huang H. Inflammation and aging: signaling pathways and intervention therapies. Signal Transduct Target Ther. (2023) 8:239. doi: 10.1038/s41392-023-01502-8 37291105 PMC10248351

[10] de Magalhães JP. Cellular senescence in normal physiology. Science. (2024) 384:1300–1. doi: 10.1126/science.adj7050 38900869

[11] de Magalhães JP Passos JF. Stress, cell senescence and organismal ageing. Mech Ageing Dev. (2018) 170:2–9. doi: 10.1016/j.mad.2017.07.001 28688962

[12] Tindle C Fonseca AG Taheri S Katkar GD Lee J Maity P. A living organoid biobank of patients with Crohn’s disease reveals molecular subtypes for personalized therapeutics. Cell Rep Med. (2024) 5:101748. doi: 10.1016/j.xcrm.2024.101748 39332415 PMC11513829

[13] Chakravarti D Lee R Multani AS Santoni A Keith Z Hsu WH. Telomere dysfunction instigates inflammation in inflammatory bowel disease. Proc Natl Acad Sci U S A. (2021) 118:e2024853118. doi: 10.1073/pnas.2024853118 34253611 PMC8307535

[14] Pawelec G. Age and immunity: what is “immunosenescence. Exp Gerontol. (2018) 105:4–9. doi: 10.1016/j.exger.2017.10.024 29111233

[15] Thomas R Wang W Su DM. Contributions of age-related thymic involution to immunosenescence and inflammaging. Immun Ageing. (2020) 17:2. doi: 10.1186/s12979-020-0173-8 31988649 PMC6971920

[16] Liu Z Liang Q Ren Y Guo C Ge X Wang L. Immunosenescence: molecular mechanisms and diseases. Signal Transduct Target Ther. (2023) 8:200. doi: 10.1038/s41392-023-01451-2 37179335 PMC10182360

[17] Singh S Poulsen GJ Bisgaard TH Bonfils L Jess T. Epidemiology of elderly onset IBD: A nationwide population-based cohort study. Clin Gastroenterol Hepatol. (2024) 23(7):1204–15.e11. doi: 10.1016/j.cgh.2024.08.011 PMC12186701 39209204

[18] Basso PJ Fonseca MT Bonfá G Alves VB Sales-Campos H Nardini V. Association among genetic predisposition, gut microbiota, and host immune response in the etiopathogenesis of inflammatory bowel disease. Braz J Med Biol Res = Rev Bras pesquisas medicas e biologicas. (2014) 47:727–37. doi: 10.1590/1414-431x20143932 PMC4143199 25075576

[19] Saez A Herrero-Fernandez B Gomez-Bris R Sánchez-Martinez H Gonzalez-Granado JM. Pathophysiology of inflammatory bowel disease: innate immune system. Int J Mol Sci. (2023) 24:1526. doi: 10.3390/ijms24021526 36675038 PMC9863490

[20] Guo Z Wang G Wu B Chou WC Cheng L Zhou C. DCAF1 regulates Treg senescence via the ROS axis during immunological aging. J Clin Invest. (2020) 130:5893–908. doi: 10.1172/JCI136466 PMC7598062 32730228

[21] Hong SJ Katz S. The elderly IBD patient in the modern era: changing paradigms in risk stratification and therapeutic management. Therap Adv Gastroenterol. (2021) 14:17562848211023399. doi: 10.1177/17562848211023399 PMC8255562 34276809

[22] Hong SJ Galati J Katz S. Crohn’s disease of the elderly: unique biology and therapeutic efficacy and safety. Gastroenterol Clin North Am. (2022) 51:425–40. doi: 10.1016/j.gtc.2021.12.014 35595423

[23] Barrett T Troup DB Wilhite SE Ledoux P Rudnev D Evangelista C. NCBI GEO: mining tens of millions of expression profiles–database and tools update. Nucleic Acids Res. (2007) 35:D760–765. doi: 10.1093/nar/gkl887 PMC1669752 17099226

[24] Verstockt S De Hertogh G van der Goten J Verstockt B Vancamelbeke M Machiels K. Gene and Mirna regulatory networks during different stages of Crohn’s disease. J Crohn’s colitis. (2019) 13:916–30. doi: 10.1093/ecco-jcc/jjz007 30657881

[25] Keir ME Fuh F Ichikawa R Acres M Hackney JA Hulme G. Regulation and role of αE integrin and gut homing integrins in migration and retention of intestinal lymphocytes during inflammatory bowel disease. J Immunol (Baltimore Md.: 1950). (2021) 207:2245–54. doi: 10.4049/jimmunol.2100220 PMC8525869 34561227

[26] Stelzer G Rosen N Plaschkes I Zimmerman S Twik M Fishilevich S. The geneCards suite: from gene data mining to disease genome sequence analyses. Curr Protoc Bioinf. (2016) 54:1.30.1–1.30.33. doi: 10.1002/cpbi.5 27322403

[27] Ritchie ME Phipson B Wu D Hu Y Law CW Shi W. limma powers differential expression analyses for RNA-sequencing and microarray studies. Nucleic Acids Res. (2015) 43:e47. doi: 10.1093/nar/gkv007 25605792 PMC4402510

[28] Subramanian A Tamayo P Mootha VK Mukherjee S Ebert BL Gillette MA. Gene set enrichment analysis: a knowledge-based approach for interpreting genome-wide expression profiles. Proc Natl Acad Sci U S A. (2005) 102:15545–50. doi: 10.1073/pnas.0506580102 PMC1239896 16199517

[29] Yu G. Gene ontology semantic similarity analysis using GOSemSim. Methods Mol Biol (Clifton N.J.). (2020) 2117:207–15. doi: 10.1007/978-1-0716-0301-7_11 31960380

[30] Kanehisa M Goto S. KEGG: kyoto encyclopedia of genes and genomes. Nucleic Acids Res. (2000) 28:27–30. doi: 10.1093/nar/28.1.27 10592173 PMC102409

[31] Langfelder P Horvath S. WGCNA: an R package for weighted correlation network analysis. BMC Bioinf. (2008) 9:559. doi: 10.1186/1471-2105-9-559 PMC2631488 19114008

[32] Davis AP Wiegers TC Johnson RJ Sciaky D Wiegers J Mattingly CJ. Comparative toxicogenomics database (CTD): update 2023. Nucleic Acids Res. (2023) 51:D1257–62. doi: 10.1093/nar/gkac833 PMC9825590 36169237

[33] Huang J Zheng W Zhang P Lin Q Chen Z Xuan J. ChIPBase v3.0: the encyclopedia of transcriptional regulations of non-coding RNAs and protein-coding genes. Nucleic Acids Res. (2023) 51:D46–56. doi: 10.1093/nar/gkac1067 PMC9825553 36399495

[34] Zhang Q Liu W Zhang HM Xie GY Miao YR Xia M. hTFtarget: A comprehensive database for regulations of human transcription factors and their targets. Genomics Proteomics Bioinf. (2020) 18:120–8. doi: 10.1016/j.gpb.2019.09.006 PMC7647694 32858223

[35] Li JH Liu S Zhou H Qu LH Yang JH. starBase v2.0: decoding miRNA-ceRNA, miRNA-ncRNA and protein-RNA interaction networks from large-scale CLIP-Seq data. Nucleic Acids Res. (2014) 42:D92–97. doi: 10.1093/nar/gkt1248 PMC3964941 24297251

[36] Sanz H Valim C Vegas E Oller JM Reverter F. SVM-RFE: selection and visualization of the most relevant features through non-linear kernels. BMC Bioinf. (2018) 19:432. doi: 10.1186/s12859-018-2451-4 PMC6245920 30453885

[37] Liu Y Zhao H. Variable importance-weighted random forests. Quantitative Biol (Beijing China). (2017) 5:338–51. doi: 10.1007/s40484-017-0121-6 PMC6051549 30034909

[38] Cai W van der Laan M. Nonparametric bootstrap inference for the targeted highly adaptive least absolute shrinkage and selection operator (LASSO) estimator. Int J Biostat. (2020) 16(2):20170070. doi: 10.1515/ijb-2017-0070 32772002

[39] Hänzelmann S Castelo R Guinney J. GSVA: gene set variation analysis for microarray and RNA-seq data. BMC Bioinf. (2013) 14:7. doi: 10.1186/1471-2105-14-7 PMC3618321 23323831

[40] Charoentong P Finotello F Angelova M Mayer C Efremova M Rieder D. Pan-cancer immunogenomic analyses reveal genotype-immunophenotype relationships and predictors of response to checkpoint blockade. Cell Rep. (2017) 18:248–62. doi: 10.1016/j.celrep.2016.12.019 28052254

[41] Chen B Khodadoust MS Liu CL Newman AM Alizadeh AA. Profiling tumor infiltrating immune cells with CIBERSORT. Methods Mol Biol. (2018) 1711:243–59. doi: 10.1007/978-1-4939-7493-1_12 PMC5895181 29344893

[42] Dolinger M Torres J Vermeire S. Crohn’s disease. Lancet. (2024) 403:1177–91. doi: 10.1016/S0140-6736(23)02586-2 38437854

[43] Salas A Hernandez-Rocha C Duijvestein M Faubion W McGovern D Vermeire S. JAK-STAT pathway targeting for the treatment of inflammatory bowel disease. Nat Rev Gastroenterol Hepatol. (2020) 17:323–37. doi: 10.1038/s41575-020-0273-0 32203403

[44] Nayar S Morrison JK Giri M Gettler K Chuang LS Walker LA. A myeloid-stromal niche and gp130 rescue in NOD2-driven Crohn’s disease. Nature. (2021) 593:275–81. doi: 10.1038/s41586-021-03484-5 PMC8284870 33789339

[45] Xu J Wakai M Xiong K Yang Y Prabakaran A Wu S. The pro-inflammatory cytokine IL6 suppresses mitochondrial function via the gp130-JAK1/STAT1/3-HIF1α/ERRα axis. Cell Rep. (2025) 44:115403. doi: 10.1016/j.celrep.2025.115403 40056415

[46] Yao H Rahman I. Perspectives on translational and therapeutic aspects of SIRT1 in inflammaging and senescence. Biochem Pharmacol. (2012) 84:1332–9. doi: 10.1016/j.bcp.2012.06.031 PMC3482299 22796566

[47] Yeung F Hoberg JE Ramsey CS Keller MD Jones DR Frye RA. Modulation of NF-kappaB-dependent transcription and cell survival by the SIRT1 deacetylase. EMBO J. (2004) 23:2369–80. doi: 10.1038/sj.emboj.7600244 PMC423286 15152190

[48] Vernier M Bourdeau V Gaumont-Leclerc MF Moiseeva O Bégin V Saad F. Regulation of E2Fs and senescence by PML nuclear bodies. Genes Dev. (2011) 25:41–50. doi: 10.1101/gad.1975111 21205865 PMC3012935

[49] Suryadevara V Hudgins AD Rajesh A Pappalardo A Karpova A Dey AK. SenNet recommendations for detecting senescent cells in different tissues. Nat Rev Mol Cell Biol. (2024) 25(12):1001–23. doi: 10.1038/s41580-024-00738-8 PMC11578798 38831121

[50] Amatullah H Fraschilla I Digumarthi S Huang J Adiliaghdam F Bonilla G. Epigenetic reader SP140 loss of function drives Crohn’s disease due to uncontrolled macrophage topoisomerases. Cell. (2022) 185:3232–47.e18. doi: 10.1016/j.cell.2022.06.048 35952671 PMC9442451

[51] Broderick L Hoffman HM. IL-1 and autoinflammatory disease: biology, pathogenesis and therapeutic targeting. Nat Rev Rheumatol. (2022) 18:448–63. doi: 10.1038/s41584-022-00797-1 PMC9210802 35729334

[52] Menghini P Corridoni D Buttó LF Osme A Shivaswamy S Lam M. Neutralization of IL-1α ameliorates Crohn’s disease-like ileitis by functional alterations of the gut microbiome. Proc Natl Acad Sci U S A. (2019) 116:26717–26. doi: 10.1073/pnas.1915043116 PMC6936591 31843928

[53] van der Pouw Kraan CT Baggen JM van Bodegraven AA Mulder CJ Zwiers A Geerts D. Defective IL-1A expression in patients with Crohn’s disease is related to attenuated MAP3K4 signaling. Hum Immunol. (2012) 73:912–9. doi: 10.1016/j.humimm.2012.06.004 22732089

[54] Park MD Le Berichel J Hamon P Wilk CM Belabed M Yatim N. Hematopoietic aging promotes cancer by fueling IL-1α-driven emergency myelopoiesis. Science. (2024) 386:eadn0327. doi: 10.1126/science.adn0327 39236155 PMC7616710

[55] Hänggi K Li J Gangadharan A Liu X Celias DP Osunmakinde O. Interleukin-1α release during necrotic-like cell death generates myeloid-driven immunosuppression that restricts anti-tumor immunity. Cancer Cell. (2024) 42:2015–31.e11. doi: 10.1016/j.ccell.2024.10.014 39577420 PMC11631672

[56] Martínez-Zamudio RI Robinson L Roux PF Bischof O. SnapShot: cellular senescence pathways. Cell. (2017) 170:816–816.e1. doi: 10.1016/j.cell.2017.07.049 28802049

[57] Deng X Shang L Du M Yuan L Xiong L Xie X. Mechanism underlying the significant role of the miR-4262/SIRT1 axis in children with inflammatory bowel disease. Exp Ther Med. (2020) 20:2227–35. doi: 10.3892/etm.2020.8918 PMC7401569 32765699

[58] Loftus EV Jr. Panés J Lacerda AP Peyrin-Biroulet L D’Haens G Panaccione R. Upadacitinib induction and maintenance therapy for Crohn’s disease. N Engl J Med. (2023) 388:1966–80. doi: 10.1056/NEJMoa2212728 37224198

[59] Murray PJ. Understanding and exploiting the endogenous interleukin-10/STAT3-mediated anti-inflammatory response. Curr Opin Pharmacol. (2006) 6:379–86. doi: 10.1016/j.coph.2006.01.010 16713356

[60] Brand S. Crohn’s disease: Th1, Th17 or both? The change of a paradigm: new immunological and genetic insights implicate Th17 cells in the pathogenesis of Crohn’s disease. Gut. (2009) 58:1152–67. doi: 10.1136/gut.2008.163667 19592695

[61] Shouval DS Biswas A Goettel JA McCann K Conaway E Redhu NS. Interleukin-10 receptor signaling in innate immune cells regulates mucosal immune tolerance and anti-inflammatory macrophage function. Immunity. (2014) 40:706–19. doi: 10.1016/j.immuni.2014.03.011 PMC4513358 24792912

[62] Ma YF Lu Y Wu Q Lou YJ Yang M Xu JY. Oral arsenic and retinoic acid for high-risk acute promyelocytic leukemia. J Hematol Oncol. (2022) 15:148. doi: 10.1186/s13045-022-01368-3 36258250 PMC9578225

[63] Zhao X Yang W Yu T Yu Y Cui X Zhou Z. Th17 cell-derived amphiregulin promotes colitis-associated intestinal fibrosis through activation of mTOR and MEK in intestinal myofibroblasts. Gastroenterology. (2023) 164:89–102. doi: 10.1053/j.gastro.2022.09.006 36113570 PMC9772145

[64] Gonzalez CG Mills RH Zhu Q Sauceda C Knight R Dulai PS. Location-specific signatures of Crohn’s disease at a multi-omics scale. Microbiome. (2022) 10:133. doi: 10.1186/s40168-022-01331-x 35999575 PMC9400277

[65] Preto AJ Chanana S Ence D Healy MD Domingo-Fernández D West KA. Multi-omics data integration identifies novel biomarkers and patient subgroups in inflammatory bowel disease. J Crohn’s colitis. (2025) 19. doi: 10.1093/ecco-jcc/jjae197 PMC11792892 39756419

[66] Garrido-Trigo A Corraliza AM Veny M Dotti I Melón-Ardanaz E Rill A. Macrophage and neutrophil heterogeneity at single-cell spatial resolution in human inflammatory bowel disease. Nat Commun. (2023) 14:4506. doi: 10.1038/s41467-023-40156-6 37495570 PMC10372067

[67] Martinez-Medina M Garcia-Gil LJ. Escherichia coli in chronic inflammatory bowel diseases: An update on adherent invasive Escherichia coli pathogenicity. World J Gastrointest Pathophysiol. (2014) 5:213–27. doi: 10.4291/wjgp.v5.i3.213 PMC4133521 25133024

[68] Davis WC. On deaf ears, Mycobacterium avium paratuberculosis in pathogenesis Crohn’s and other diseases. World J Gastroenterol. (2015) 21:13411–7. doi: 10.3748/wjg.v21.i48.13411 PMC4690169 26730151

[69] Yang H Wang T Qian C Wang H Yu D Shi M. Gut microbial-derived phenylacetylglutamine accelerates host cellular senescence. Nat Aging. (2025) 5:401–18. doi: 10.1038/s43587-024-00795-w 39794469

[70] Nielsen CM White MJ Goodier MR Riley EM. Functional significance of CD57 expression on human NK cells and relevance to disease. Front Immunol. (2013) 4:422. doi: 10.3389/fimmu.2013.00422 24367364 PMC3856678

[71] Zhou D Borsa M Simon AK. Hallmarks and detection techniques of cellular senescence and cellular ageing in immune cells. Aging Cell. (2021) 20:e13316. doi: 10.1111/acel.13316 33524238 PMC7884036

