# Supplementary material for: Cellular senescence defining the disease characteristics of Crohn’s disease
Source: Front Immunol. 2025 Jun 30;16:1616531. doi: 10.3389/fimmu.2025.1616531 (PMC12256553; doi:10.3389/fimmu.2025.1616531)
Supplement: Supplementary file 1 [file DataSheet1.docx]

**Supplementary figures**


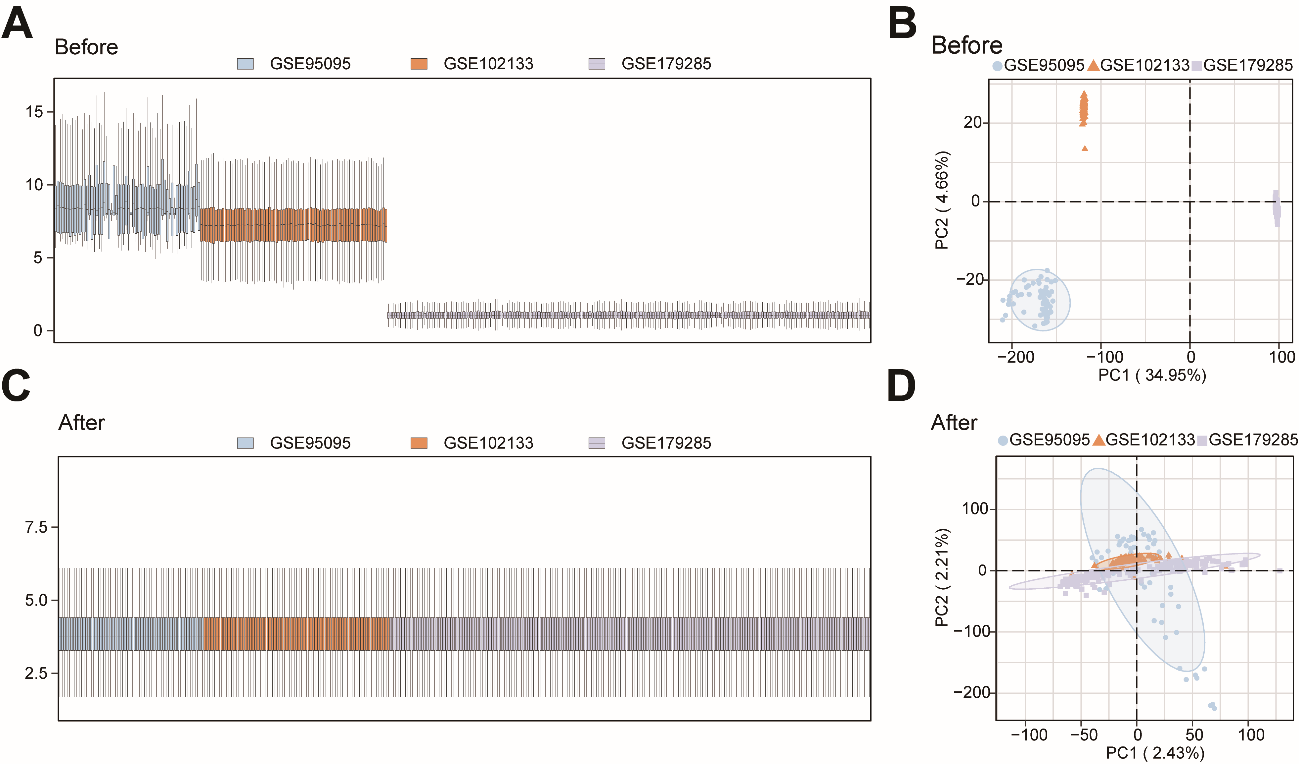


**Figure S1. Processing of datasets (GSE11691, GSE25628, and GSE86534).**

(**A**) Box plot of the combined dataset before correction. The colors represent corresponding samples from different datasets.

(**B**) PCA (principal component analysis) plot of the combined dataset before correction. The dots in the graph represent samples, and the colors represent corresponding samples from different datasets.

(**C**) Box plot of the combined dataset after correction. The colors represent corresponding samples from different datasets.

(**D**) The PCA plot of the combined dataset after correction. The dots in the graph represent samples, and the colors represent corresponding samples from different datasets.


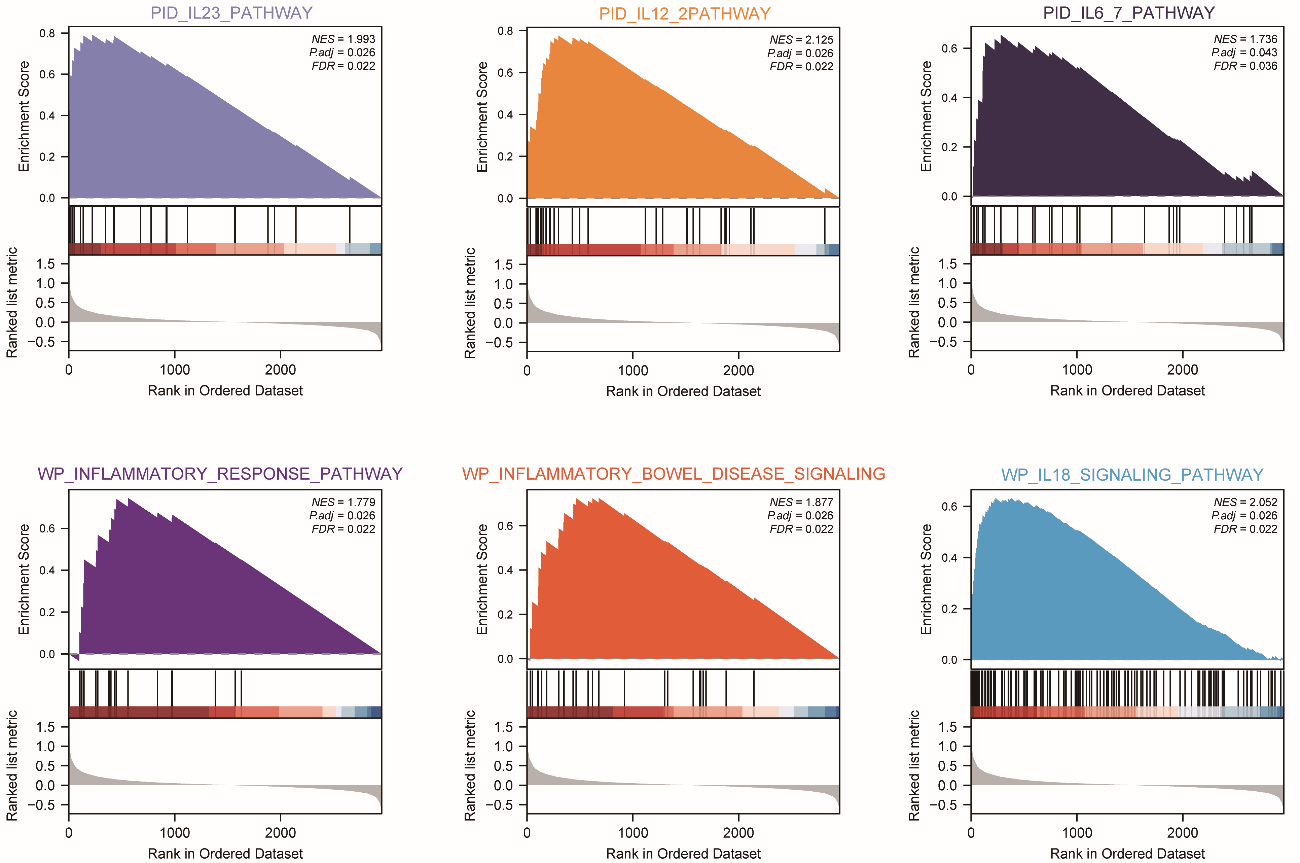


**Figure 2. GSEA enrichment analysis.** GSEA demonstrated significant enrichment of DEGs (CD vs Control) in pathways, including PID_IL23_PATHWAY, PID_IL12_2PATHWAY, PID_IL6_7_PATHWAY, WP_INFLAMMATORY_BOWEL_DISEASE_SIGNALING, WP_INFLAMMATORY_RESPONSE_PATHWAY, WP_IL18_SIGNALING_PATHWAY.


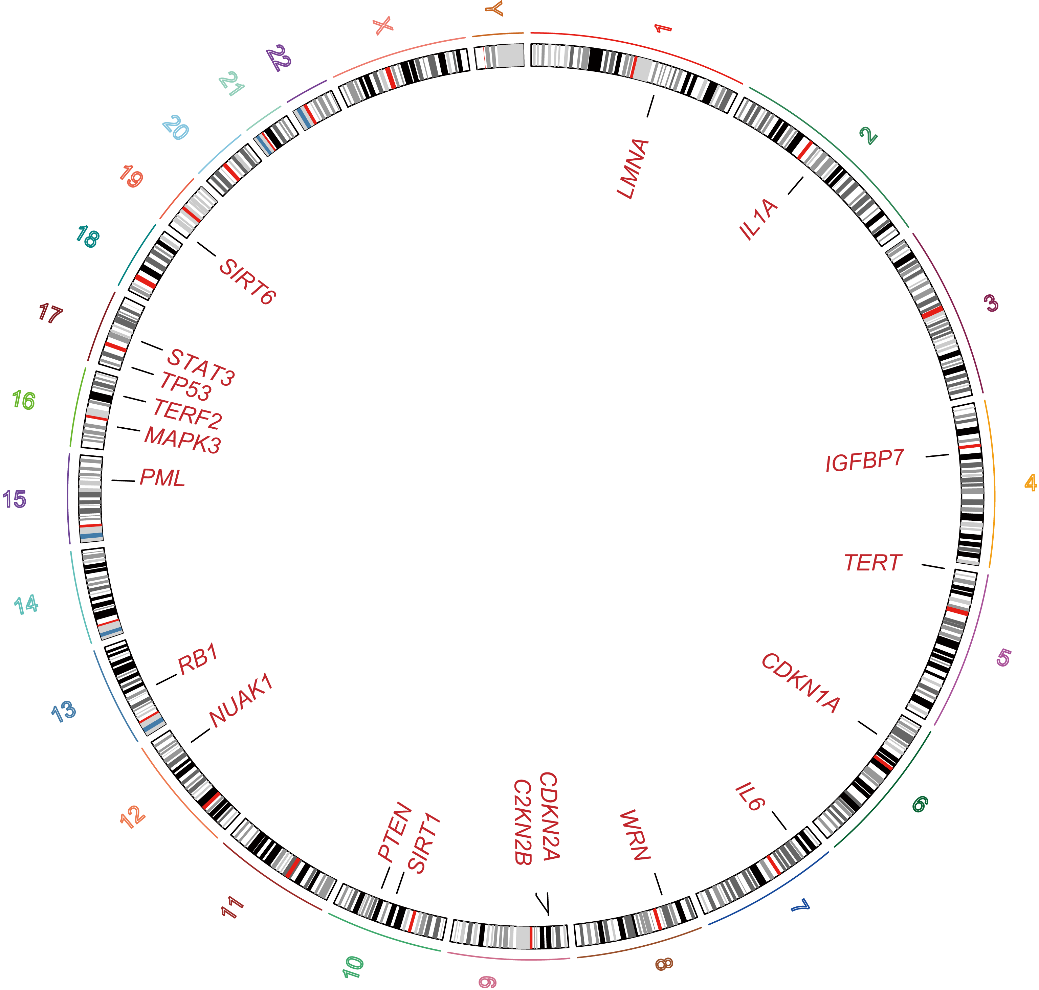


**Figure 3. The location of 19 cellular senescence-related DEGs on chromosomes.**


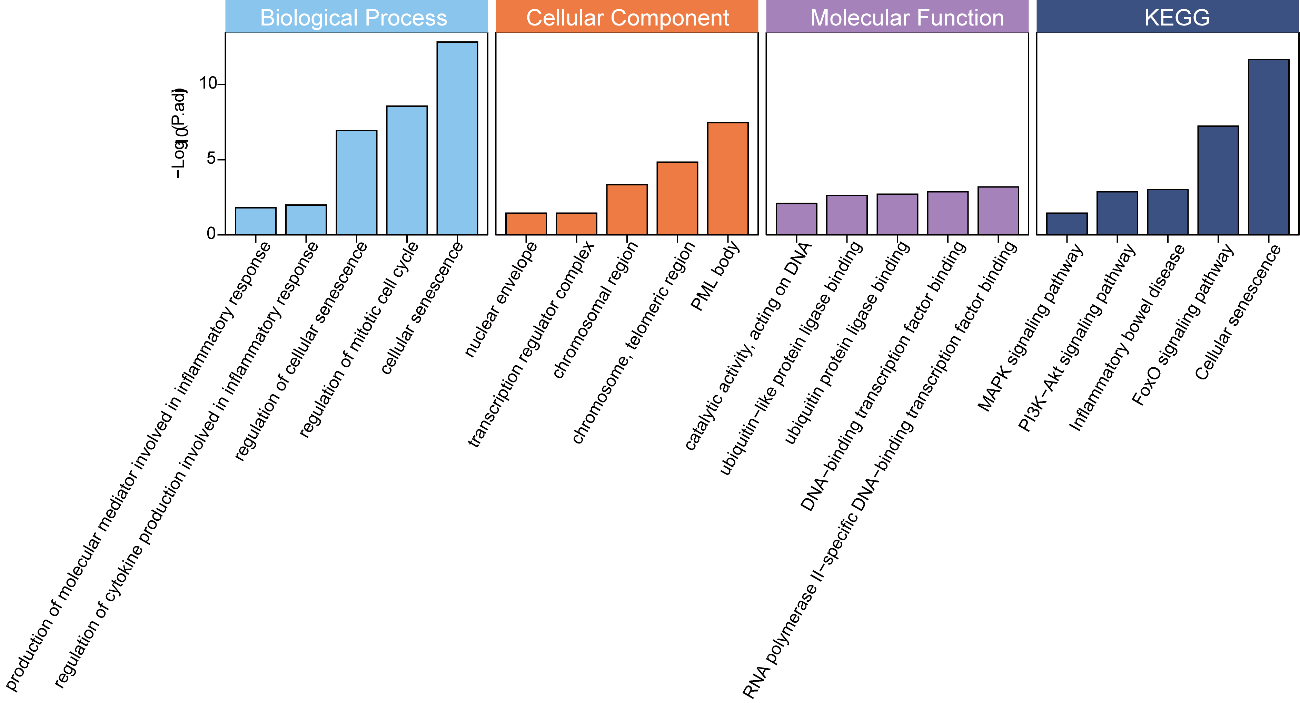


**Figure S4.** **The bar chart shows GO and KEGG analysis for CSRDEGs.**


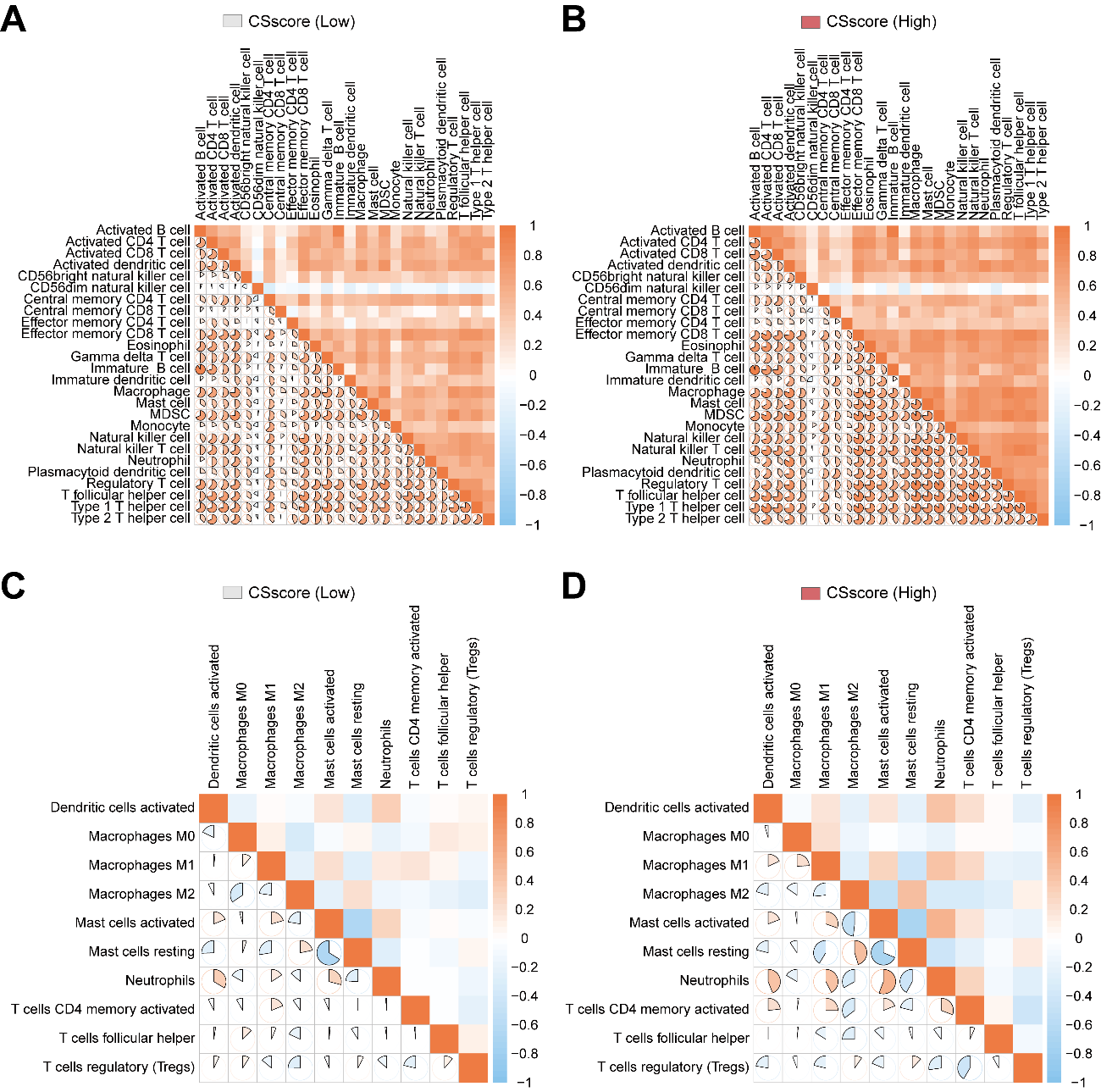


**Figure S5. The correlation of differentially infiltrated immune cells in CSscore (Low or High).**

(**A**) A heatmap showing the correlation of differentially infiltrated immune cells (26 types) in CSscore (Low).

(**B**) A heatmap showing the correlation of differentially infiltrated immune cells (26 types) in CSscore (High).

(**C**) A heatmap showing the correlation of differentially infiltrated immune cells (10 types) in CSscore (Low).

(**D**) A heatmap showing the correlation of differentially infiltrated immune cells (10 types) in CSscore (High).


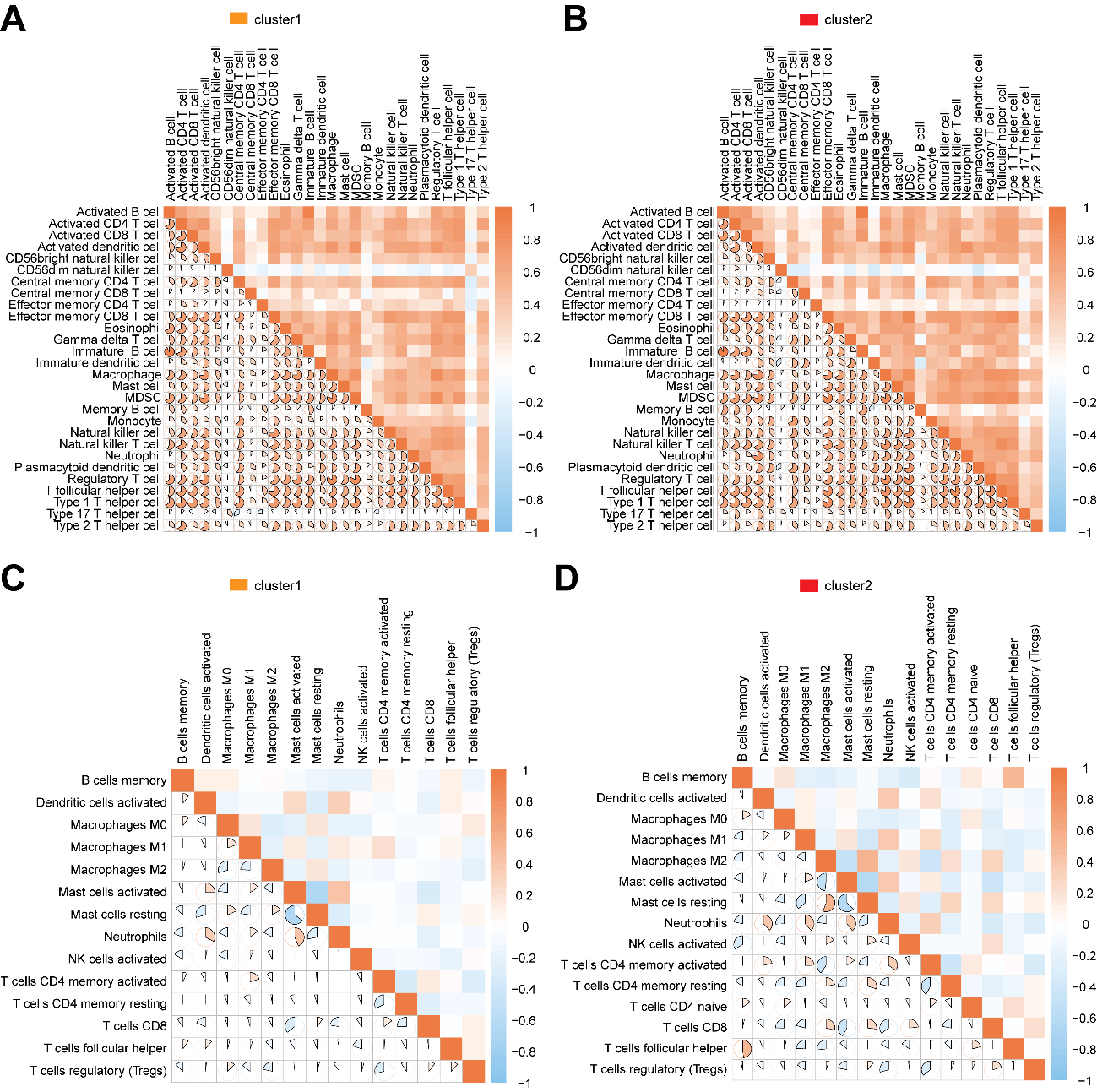


**Figure S6. The correlation of differentially infiltrated immune cells in CD Subtypes.**

(**A**) A heatmap showing the correlation of differentially infiltrated immune cells (28 types) in cluster 1.

(**B**) A heatmap showing the correlation of differentially infiltrated immune cells (28 types) in cluster 2.

(**C**) A heatmap showing the correlation of differentially infiltrated immune cells (15 types) in cluster 1.

(**D**) A heatmap showing the correlation of differentially infiltrated immune cells (15 types) in cluster 2.
