# Supplementary material for: Cellular senescence defining the disease characteristics of Crohn’s disease
Source: Front Immunol. 2025 Jun 30;16:1616531. doi: 10.3389/fimmu.2025.1616531 (PMC12256553; doi:10.3389/fimmu.2025.1616531)
Supplement: Supplementary file 2 [file Table1.docx]

**Supplementary Table****s**

**Table S1. Crohn’s disease Dataset Information list.**

|  | **GSE95095** | **GSE102133** | **GSE179285** |
| --- | --- | --- | --- |
| Platform | GPL14951 | GPL6244 | GPL6480 |
| Species | Homo sapiens | Homo sapiens | Homo sapiens |
| Experiment type | Expression profiling by array | Expression profiling by array | Expression profiling by array |
| Tissue | intestinal tissue | intestinal tissue | intestinal tissue |
| Samples in Control group | 12 | 12 | 31 |
| Samples in CD group | 48 | 65 | 168 |
| Reference | \ | Gene and Mirna Regulatory Networks During Different Stages of Crohn's Disease | Regulation and Role of αE Integrin and Gut Homing Integrins in Migration and Retention of Intestinal Lymphocytes during Inflammatory Bowel Disease |

CD：Crohn’s disease.

**Table S2. List of Cellular senescence related genes (CSRGs).**

| **Gene Symbol** | **Full name** |  | **Gene Symbol** | **Full name** |
| --- | --- | --- | --- | --- |
| *TP53* | Tumor Protein P53 | | *TBX2* | T-Box Transcription Factor 2 |
| *CDKN2A* | Cyclin Dependent Kinase Inhibitor 2A | | *WRN* | WRN RecQ Like Helicase |
| *CDKN1A* | Cyclin Dependent Kinase Inhibitor 1A | | *CGAS* | Cyclic GMP-AMP Synthase |
| *SIRT1* | Sirtuin 1 |  | *PTEN* | Phosphatase And Tensin Homolog |
| *RSL1D1* | Ribosomal L1 Domain Containing 1 | | *YPEL3* | Yippee Like 3 |
| *TERT* | Telomerase Reverse Transcriptase | | *H2AX* | H2A.X Variant Histone |
| *RB1* | RB Transcriptional Corepressor 1 | | *HMGA2* | High Mobility Group AT-Hook 2 |
| *PML* | PML Nuclear Body Scaffold | | *PHB1* | Prohibitin 1 |
| *SIRT6* | Sirtuin 6 |  | *JUN* | Jun Proto-Oncogene, AP-1 Transcription Factor Subunit |
| *MAPK14* | Mitogen-Activated Protein Kinase 14 | | *NFKB1* | Nuclear Factor Kappa B Subunit 1 |
| *ATM* | ATM Serine/Threonine Kinase | | *IGFBP7* | Insulin Like Growth Factor Binding Protein 7 |
| *TERF2* | Telomeric Repeat Binding Factor 2 | | *POT1* | Protection Of Telomeres 1 |
| *BMI1* | BMI1 Proto-Oncogene, Polycomb Ring Finger | | *TBX3* | T-Box Transcription Factor 3 |
| *CDK2* | Cyclin Dependent Kinase 2 | | *STAT3* | Signal Transducer And Activator Of Transcription 3 |
| *CDKN1B* | Cyclin Dependent Kinase Inhibitor 1B | | *MAPK3* | Mitogen-Activated Protein Kinase 3 |
| *E2F1* | E2F Transcription Factor 1 | | *CDKN2B* | Cyclin Dependent Kinase Inhibitor 2B |
| *LMNA* | Lamin A/C | | *CXCL8* | C-X-C Motif Chemokine Ligand 8 |
| *EZH2* | Enhancer Of Zeste 2 Polycomb Repressive Complex 2 Subunit | | *MYC* | MYC Proto-Oncogene, BHLH Transcription Factor |
| *CDK4* | Cyclin Dependent Kinase 4 | | *HIRA* | Histone Cell Cycle Regulator |
| *ID1* | Inhibitor Of DNA Binding 1 | | *RELA* | RELA Proto-Oncogene, NF-KB Subunit |
| *CDK6* | Cyclin Dependent Kinase 6 | | *KDM6B* | Lysine Demethylase 6B |
| *MDM2* | MDM2 Proto-Oncogene | | *ZNF277* | Zinc Finger Protein 277 |
| *MAPK1* | Mitogen-Activated Protein Kinase 1 | | *IL1A* | Interleukin 1 Alpha |
| *IL6* | Interleukin 6 | | *TERF1* | Telomeric Repeat Binding Factor 1 |
| *SP1* | Sp1 Transcription Factor | | *CCNA2* | Cyclin A2 |
| *NUAK1* | NUAK Family Kinase 1 | | *H1-4* | H1.4 Linker Histone, Cluster Member |
| *UBN1* | Ubinuclein 1 | | *FOS* | Fos Proto-Oncogene, AP-1 Transcription Factor Subunit |
| *LMNB1* | Lamin B1 |  |  |  |

**Table S3. GSEA enrichment analysis.**

| **ONTOLOGY** | **ID** | **Description** | **pvalue** | **p.adjust** | **qvalue** |
| --- | --- | --- | --- | --- | --- |
| BP | GO:0090398 | Cellular senescence | 8.63E-17 | 1.55E-13 | 5.61E-14 |
| BP | GO:0007346 | Regulation of mitotic cell cycle | 7.74E-12 | 2.77E-09 | 1.01E-09 |
| BP | GO:2000772 | Regulation of cellular senescence | 1.1E-09 | 1.16E-07 | 4.22E-08 |
| BP | GO:1900015 | Regulation of cytokine production involved in Inflammatory response | 0.00257 | 0.010343 | 0.003757 |
| BP | GO:0002532 | production of molecular mediator involved in inflammatory response | 0.004255 | 0.015681 | 0.005696 |
| CC | GO:0016605 | PML body | 5.25E-10 | 3.52E-08 | 1.83E-08 |
| CC | GO:0000781 | Chromosome, telomeric region | 4.34E-07 | 1.45E-05 | 7.54E-06 |
| CC | GO:0098687 | Chromosomal region | 2.07E-05 | 0.000463 | 0.00024 |
| CC | GO:0005635 | Nuclear envelope | 0.010512 | 0.036019 | 0.018674 |
| CC | GO:0005667 | Transcription regulator complex | 0.010752 | 0.036019 | 0.018674 |
| MF | GO:0061629 | RNA polymerase II-specific DNA-binding transcription factor binding | 2.19E-05 | 0.000645 | 0.000282 |
| MF | GO:0140297 | DNA-binding transcription factor binding | 9.18E-05 | 0.001349 | 0.000589 |
| MF | GO:0031625 | Ubiquitin protein ligase binding | 0.000215 | 0.001977 | 0.000864 |
| MF | GO:0044389 | Ubiquitin-like protein ligase binding | 0.000273 | 0.002357 | 0.001029 |
| MF | GO:0140097 | Catalytic activity, acting on DNA | 0.001453 | 0.008217 | 0.003589 |
| KEGG | hsa04218 | Cellular senescence | 1.34E-14 | 2.23E-12 | 1.08E-12 |
| KEGG | hsa04068 | FoxO signaling pathway | 1.35E-09 | 5.92E-08 | 2.85E-08 |
| KEGG | hsa05321 | Inflammatory bowel disease | 0.000205 | 0.000971 | 0.000468 |
| KEGG | hsa04151 | PI3K-Akt signaling pathway | 0.000312 | 0.001364 | 0.000657 |
| KEGG | hsa04010 | MAPK signaling pathway | 0.015247 | 0.036157 | 0.017425 |

**Table S4. GO and KEGG enrichment analysis results of CSRDEGs.**

| **ONTOLOGY** | **ID** | **Description** | **GeneRatio** | | **BgRatio** | **p.adjust** | **qvalue** |
| --- | --- | --- | --- | --- | --- | --- | --- |
| BP | GO:0034976 | Response to Cellular senescence | 12/13 | | 257/18800 | 3.83E-19 | 1.88E-19 |
| BP | GO:0030968 | Endoplasmic reticulum unfolded protein response | 9/13 | | 76/18800 | 5.64E-17 | 2.77E-17 |
| BP | GO:0070059 | Intrinsic apoptotic signaling pathway in response to Cellular senescence | 8/13 | | 63/18800 | 2.3E-15 | 1.13E-15 |
| BP | GO:0097193 | Intrinsic apoptotic signaling pathway | 8/13 | | 295/18800 | 2.6E-10 | 1.28E-10 |
| BP | GO:1905897 | Regulation of response to Cellular senescence | 6/13 | | 82/18800 | 5.41E-10 | 2.66E-10 |
| CC | GO:0090575 | RNA polymerase II transcription regulator complex | 7/13 | | 230/19594 | 2.31E-09 | 1.29E-09 |
| CC | GO:0005667 | Transcription regulator complex | 7/13 | | 483/19594 | 2.04E-07 | 1.14E-07 |
| MF | GO:0046982 | Protein heterodimerization activity | 7/13 | | 332/18410 | 4.46E-08 | 1.15E-08 |
| MF | GO:0001228 | DNA-binding transcription activator activity, RNA polymerase II-specific | 6/13 | | 462/18410 | 6.14E-06 | 1.58E-06 |
| MF | GO:0001216 | DNA-binding transcription activator activity | 6/13 | | 466/18410 | 6.14E-06 | 1.58E-06 |
| MF | GO:0140297 | DNA-binding transcription factor binding | 4/13 | | 470/18410 | 0.001359 | 0.00035 |
| MF | GO:0061629 | RNA polymerase II-specific DNA-binding transcription factor binding | 3/13 | | 348/18410 | 0.006793 | 0.001751 |
| KEGG | hsa04141 | Protein processing in endoplasmic reticulum | 9/11 | | 171/8164 | 3.77E-12 | 1.76E-12 |
| KEGG | hsa04210 | Apoptosis | | 6/11 | 136/8164 | 3.01E-07 | 1.4E-07 |
| KEGG | hsa04668 | TNF signaling pathway | | 4/11 | 112/8164 | 0.000116 | 5.41E-05 |
| KEGG | hsa04010 | MAPK signaling pathway | | 3/11 | 294/8164 | 0.019859 | 0.00925 |
| KEGG | hsa04657 | IL-17 signaling pathway | | 2/11 | 94/8164 | 0.020593 | 0.009591 |

**Table S5. Information on of hub gene.**

| **Gene Symbol** | **Full name** |
| --- | --- |
| *CDKN1A* | Cyclin Dependent Kinase Inhibitor 1A |
| *CDKN2B* | Cyclin Dependent Kinase Inhibitor 2B |
| *IGFBP7* | Insulin Like Growth Factor Binding Protein 7 |
| *IL1A* | Interleukin 1 Alpha |
| *IL6* | Interleukin 6 |
| *MAPK3* | Mitogen-Activated Protein Kinase 3 |
| *PML* | PML Nuclear Body Scaffold |
| *SIRT1* | Sirtuin 1 |
| *SIRT6* | Sirtuin 6 |
| *STAT3* | Signal Transducer And Activator Of Transcription 3 |

**Table S6. mRNA-drug interaction network nodes.**

| **mRNA** | **drug** |  | **mRNA** | **drug** |
| --- | --- | --- | --- | --- |
| *IL6* | Lipopolysaccharides | | *IL6* | Tobacco Smoke Pollution |
| *MAPK3* | Lipopolysaccharides | | *MAPK3* | sodium arsenite |
| *IL6* | Dextran Sulfate | | *IL6* | Bleomycin |
| *IL6* | lipopolysaccharide, Escherichia coli O111 B4 | | *IL6* | Doxorubicin |
| *IL6* | deoxynivalenol | | *IL6* | Mustard Gas |
| *IL6* | lipopolysaccharide, E coli O55-B5 | | *IL6* | Ovalbumin |
| *IL6* | Particulate Matter | | *IL6* | Quercetin |
| *MAPK3* | lipopolysaccharide, Escherichia coli O111 B4 | | *IL6* | Soot |
| *IL6* | Dietary Fats | | *IL6* | Tetradecanoylphorbol Acetate |
| *MAPK3* | Tetradecanoylphorbol Acetate | | *IL6* | Trinitrobenzenesulfonic Acid |
| *IL6* | Acetaminophen | | *MAPK3* | Acetaminophen |
| *IL6* | bisphenol A | | *MAPK3* | Cadmium Chloride |
| *IL6* | Ozone |  | *MAPK3* | Cisplatin |
| *SIRT1* | Resveratrol | | *MAPK3* | Estradiol |
| *IL6* | Carbon Tetrachloride | | *STAT3* | Carbon Tetrachloride |
| *IL1A* | Lipopolysaccharides | | *IL6* | 1-Naphthylisothiocyanate |
| *MAPK3* | deoxynivalenol | | *IL6* | Nanotubes, Carbon |
| *MAPK3* | bisphenol A | | *IL6* | Poly I-C |
| *IL6* | Dexamethasone | | *IL6* | Resveratrol |
| *IL6* | lipopolysaccharide, E. coli O26-B6 | | *IL6* | Silicon Dioxide |
| *IL6* | Ethanol |  | *IL6* | Sodium Glutamate |
| *IL6* | Cisplatin |  | *IL6* | T-2 Toxin |
| *MAPK3* | lipopolysaccharide, E coli O55-B5 | | *IL6* | Vehicle Emissions |
| *STAT3* | Lipopolysaccharides | | *IL6* | Zymosan |
| *IL6* | Air Pollutants | | *MAPK3* | Asbestos, Crocidolite |
| *IL6* | sodium arsenite | | *MAPK3* | Benzo(a)pyrene |
| *MAPK3* | Carbon Tetrachloride | | *MAPK3* | Cadmium |
| *MAPK3* | Hydrogen Peroxide | | *MAPK3* | Phenylephrine |
| *IL6* | Diethylnitrosamine | | *SIRT1* | Dietary Fats |
| *IL6* | Paraquat |  | *SIRT1* | Ethanol |

**Table S7. mRNA-TF interaction network nodes.**

| **mRNA** | **Transcription factor** | | **mRNA** | **Transcription factor** |
| --- | --- | --- | --- | --- |
| *CDKN1A* | EP300 |  | *PML* | GABPA |
| *IL6* | FOXA1 |  | *PML* | KLF4 |
| *IL6* | CEBPA |  | *PML* | MYB |
| *MAPK3* | FOS |  | *PML* | POLR2A |
| *MAPK3* | FOSL1 |  | *PML* | RUNX1T1 |
| *MAPK3* | GRHL3 |  | *PML* | SPI1 |
| *MAPK3* | JUN |  | *PML* | TBP |
| *MAPK3* | KLF5 |  | *PML* | ZNF263 |
| *MAPK3* | KLF9 |  | *SIRT1* | GABPA |
| *MAPK3* | MAFK |  | *SIRT1* | MAZ |
| *MAPK3* | POLR2A |  | *SIRT1* | BHLHE40 |
| *MAPK3* | RAD21 |  | *SIRT1* | BRD4 |
| *MAPK3* | SMC3 |  | *SIRT1* | E2F1 |
| *MAPK3* | TFAP2A |  | *SIRT6* | FOXA1 |
| *MAPK3* | TFAP2C |  | *SIRT6* | KLF1 |
| *PML* | CDK9 |  | *SIRT6* | KLF4 |
| *PML* | CTCF |  | *SIRT6* | KLF9 |
| *PML* | ELF1 |  | *SIRT6* | MAX |
| *PML* | EP300 |  | *SIRT6* | MAZ |
| *PML* | ERG |  | *SIRT6* | SPI1 |
| *PML* | ETS1 |  | *SIRT6* | TFAP2C |
| *PML* | FLI1 |  |  |  |

**Table S8. mRNA-miRNA interaction network nodes.**

| **mRNA** | **miRNA** |  | **mRNA** | **miRNA** |
| --- | --- | --- | --- | --- |
| *CDKN1A* | *hsa-miR-17-5p* | | *SIRT1* | *hsa-miR-135a-5p* |
| *CDKN1A* | *hsa-miR-17-5p* | | *SIRT1* | *hsa-miR-141-3p* |
| *CDKN1A* | *hsa-miR-20a-5p* | | *SIRT1* | *hsa-miR-200a-3p* |
| *CDKN1A* | *hsa-miR-20a-5p* | | *SIRT1* | *hsa-miR-135b-5p* |
| *CDKN1A* | *hsa-miR-93-5p* | | *SIRT1* | *hsa-miR-449a* |
| *CDKN1A* | *hsa-miR-93-5p* | | *SIRT1* | *hsa-miR-449a* |
| *CDKN1A* | *hsa-miR-106a-5p* | | *SIRT1* | *hsa-miR-487a-3p* |
| *CDKN1A* | *hsa-miR-106a-5p* | | *SIRT1* | *hsa-miR-543* |
| *CDKN1A* | *hsa-miR-106b-5p* | | *SIRT1* | *hsa-miR-543* |
| *CDKN1A* | *hsa-miR-106b-5p* | | *SIRT6* | *hsa-miR-33a-5p* |
| *CDKN1A* | *hsa-miR-370-3p* | | *SIRT6* | *hsa-miR-33b-5p* |
| *CDKN1A* | *hsa-miR-20b-5p* | | *STAT3* | *hsa-miR-17-5p* |
| *CDKN1A* | *hsa-miR-20b-5p* | | *STAT3* | *hsa-miR-17-5p* |
| *CDKN1A* | *hsa-miR-519d-3p* | | *STAT3* | *hsa-miR-20a-5p* |
| *CDKN1A* | *hsa-miR-519d-3p* | | *STAT3* | *hsa-miR-20a-5p* |
| *CDKN2B* | *hsa-miR-451a* | | *STAT3* | *hsa-miR-21-5p* |
| *IL1A* | *hsa-miR-24-3p* | | *STAT3* | *hsa-miR-93-5p* |
| *IL1A* | *hsa-miR-181a-5p* | | *STAT3* | *hsa-miR-93-5p* |
| *IL1A* | *hsa-miR-181b-5p* | | *STAT3* | *hsa-miR-106a-5p* |
| *IL1A* | *hsa-miR-181c-5p* | | *STAT3* | *hsa-miR-106a-5p* |
| *IL1A* | *hsa-miR-543* | | *STAT3* | *hsa-miR-124-3p* |
| *IL6* | *hsa-miR-149-5p* | | *STAT3* | *hsa-miR-125b-5p* |
| *MAPK3* | *hsa-miR-613* | | *STAT3* | *hsa-miR-130a-3p* |
| *MAPK3* | *hsa-miR-129-2-3p* | | *STAT3* | *hsa-miR-125a-5p* |
| *PML* | *hsa-miR-124-3p* | | *STAT3* | *hsa-miR-106b-5p* |
| *SIRT1* | *hsa-miR-22-3p* | | *STAT3* | *hsa-miR-106b-5p* |
| *SIRT1* | *hsa-miR-30a-5p* | | *STAT3* | *hsa-miR-301a-3p* |
| *SIRT1* | *hsa-miR-199a-5p* | | *STAT3* | *hsa-miR-130b-3p* |
| *SIRT1* | *hsa-miR-30c-5p* | | *STAT3* | *hsa-miR-20b-5p* |
| *SIRT1* | *hsa-miR-30d-5p* | | *STAT3* | *hsa-miR-20b-5p* |
| *SIRT1* | *hsa-miR-34a-5p* | | *STAT3* | *hsa-miR-519d-3p* |
| *SIRT1* | *hsa-miR-34a-5p* | | *STAT3* | *hsa-miR-519d-3p* |
| *SIRT1* | *hsa-miR-199b-5p* | | *STAT3* | *hsa-miR-506-3p* |
| *SIRT1* | *hsa-miR-212-3p* | | *STAT3* | *hsa-miR-590-5p* |
| *SIRT1* | *hsa-miR-212-3p* | | *STAT3* | *hsa-miR-454-3p* |
| *SIRT1* | *hsa-miR-128-3p* | | *STAT3* | *hsa-miR-665* |
| *SIRT1* | *hsa-miR-132-3p* | | *STAT3* | *hsa-miR-301b-3p* |
| *SIRT1* | *hsa-miR-132-3p* | |  |  |

**Table S9. mRNA-miRNA interaction network nodes.**

| **mRNA** | **RBP** |  | **mRNA** | **RBP** |
| --- | --- | --- | --- | --- |
| *CDKN1A* | CSTF2T |  | *SIRT1* | HNRNPC |
| *CDKN1A* | DDX3X |  | *SIRT1* | IGF2BP3 |
| *CDKN1A* | ELAVL1 |  | *SIRT1* | RBMX |
| *CDKN1A* | G3BP1 |  | *SIRT1* | RNPS1 |
| *CDKN1A* | HNRNPC |  | *SIRT1* | SCAF4 |
| *CDKN1A* | PTBP1 |  | *SIRT1* | SCAF8 |
| *CDKN1A* | RBFOX2 |  | *SIRT1* | TARDBP |
| *CDKN1A* | RBMX |  | *SIRT1* | U2AF2 |
| *CDKN1A* | RNPS1 |  | *STAT3* | ALYREF |
| *CDKN1A* | TARDBP |  | *STAT3* | CELF2 |
| *CDKN2B* | ELAVL1 |  | *STAT3* | DDX3X |
| *CDKN2B* | UPF1 |  | *STAT3* | ELAVL1 |
| *IGFBP7* | ELAVL1 |  | *STAT3* | G3BP1 |
| *IGFBP7* | HNRNPC |  | *STAT3* | HNRNPC |
| *MAPK3* | RBFOX2 |  | *STAT3* | IGF2BP1 |
| *MAPK3* | SCAF4 |  | *STAT3* | RBFOX2 |
| *MAPK3* | SCAF8 |  | *STAT3* | RBMX |
| *PML* | RBMX |  | *STAT3* | RNPS1 |
| *PML* | SCAF8 |  | *STAT3* | TARDBP |
| *PML* | YTHDF1 |  | *STAT3* | U2AF1 |
| *SIRT1* | ALYREF |  | *STAT3* | U2AF2 |
| *SIRT1* | DDX3X |  | *STAT3* | UPF1 |
| *SIRT1* | ELAVL1 |  | *STAT3* | YBX1 |
| *SIRT1* | G3BP1 |  |  |  |

**Table S10. The expression of HLA genes in this study.**

| **Gene** | **logFC** | **P.Value** | **adj.P.Val** | **group** |
| --- | --- | --- | --- | --- |
| HHLA2 | -0.23736 | 0.001856 | 0.009428 | down |
| HHLA3 | -0.02066 | 0.531706 | 0.688619 | no |
| HLA-DMA | 0.198478 | 0.014056 | 0.046498 | up |
| HLA-DMA | 0.198478 | 0.014056 | 0.046498 | up |
| HLA-DMB | 0.08425 | 0.334423 | 0.500898 | no |
| HLA-DMB | 0.08425 | 0.334423 | 0.500898 | no |
| HLA-DOA | 0.217692 | 0.008545 | 0.031456 | up |
| HLA-DOA | 0.217692 | 0.008545 | 0.031456 | up |
| HLA-DOB | 0.267749 | 0.001735 | 0.00895 | up |
| HLA-DPA1 | 0.421398 | 9.14E-05 | 0.000893 | up |
| HLA-DPB1 | 0.357228 | 0.000433 | 0.003013 | up |
| HLA-DPB2 | 0.199557 | 0.005249 | 0.021459 | up |
| HLA-DPB2 | 0.199557 | 0.005249 | 0.021459 | up |
| HLA-DQB1 | 0.295614 | 0.00086 | 0.005182 | up |
| HLA-DRB5 | 0.41125 | 0.001982 | 0.009918 | up |
